# *EGFR*-mutant transformed small cell lung cancer harbors intratumoral heterogeneity targetable with MEK inhibitor combination therapy

**DOI:** 10.1172/jci.insight.197008

**Published:** 2026-01-23

**Authors:** Atsuko Ogino, Amir Vajdi, Xinmeng Jasmine Mu, Navin R. Mahadevan, Kenneth Ngo, Matthew A. Booker, Paloma Cejas, Jeffrey J. Okoro, Man Xu, Benjamin F. Springer, Benjamin K. Eschle, Cameron M. Messier, Stephen Wang, Sudeepa Syamala, Rubii M. Tamen, Anika E. Adeni, Emily S. Chambers, Israel Canadas, Tran Thai, Camilla L. Christensen, Chunxiao Xu, Patrick H. Lizotte, Geoffrey R. Oxnard, Hideo Watanabe, Henry W. Long, Prafulla C. Gokhale, Cloud P. Paweletz, Lynette M. Sholl, Matthew G. Oser, David A. Barbie, Michael Y. Tolstorukov, Pasi A. Jänne

**Affiliations:** 1 Department of Medical Oncology, Dana-Farber Cancer Institute and Harvard Medical School, Boston, Massachusetts, USA.; 2 Department of Informatics and Analytics, Dana-Farber Cancer Institute, Boston, Massachusetts, USA.; 3 Broad Institute of MIT and Harvard, Cambridge, Massachusetts, USA.; 4 Department of Pathology, Brigham and Women’s Hospital, Boston, Massachusetts, USA.; 5 Belfer Center for Applied Cancer Science,; 6 Center for Functional Cancer Epigenetics, and; 7 Experimental Therapeutics Core, Dana-Farber Cancer Institute, Boston, Massachusetts, USA.; 8 Blood Cell Development and Function Program, Fox Chase Cancer Center, Philadelphia, Pennsylvania, USA.; 9 Catherine and Henry J. Gaisman Division of Pulmonary, Critical Care, and Sleep Medicine, Department of Medicine;; 10 Tisch Cancer Institute; and; 11 Department of Genetics and Genomic Sciences, Icahn School of Medicine at Mount Sinai, New York, New York, USA.; 12 Lowe Center for Thoracic Oncology, Dana-Farber Cancer Institute, Boston, Massachusetts, USA.; 13 Parker Institute for Cancer Immunotherapy, San Francisco, California, USA.

**Keywords:** Cell biology, Oncology, Drug therapy, Lung cancer

## Abstract

Small cell lung cancer (SCLC) transformation is an incompletely characterized mechanism of resistance to epidermal growth factor receptor tyrosine kinase inhibitors (EGFR-TKIs) in *EGFR*-mutant cancers, limiting development of optimal treatment approaches. Through single-cell RNA sequencing of malignant pleural effusions from patients who underwent SCLC transformation, we identified heterogeneity and diversity, including distinct neuroendocrine (NE) and mesenchymal non-NE cancer cell subsets, which were maintained in patient-derived cell lines. We demonstrate that EZH2 regulates EGFR expression in NE cells where EGFR expression is silenced at baseline. Although neither epigenetic derepression nor exogenous overexpression of mutant EGFR sensitized the cells to EGFR inhibition, non-NE cells exhibited selective sensitivity to MEK inhibitors. Combined MEK inhibitor and chemotherapy effectively inhibited growth of both NE and non-NE cells in vitro and in vivo. Our findings demonstrate that *EGFR*-mutant SCLC is composed of mixed cell states with distinct therapeutic vulnerabilities and offer a therapeutic strategy to target tumor heterogeneity in highly plastic and treatment-resistant malignancies such as transformed SCLC.

## Abstract 2

*EGFR* mutant SCLC comprises mixed cell states with distinct therapeutic vulnerabilities, and MEK inhibitor combination therapy may offer a novel therapeutic strategy to target heterogeneity.

## Introduction

Epidermal growth factor receptor tyrosine kinase inhibitors (EGFR-TKIs) are an effective treatment for *EGFR*-mutant (*EGFR*-mt) lung adenocarcinoma (LUAD). In almost all cases, however, the response duration is limited, and resistance ultimately emerges. Resistance mechanisms to EGFR-TKIs include development of *EGFR*-resistant mutations and bypassing signaling activation, as well as a lineage switch from LUAD to small cell lung cancer (SCLC). Roughly 3%–14% of patients with *EGFR*-mt LUAD undergo SCLC transformation (1, 2), and median time to transformation is 16–17.8 months after diagnosis (3, 4). The mechanism associated with this transformation is largely unknown, highlighting the need for further understanding to develop more effective treatment strategies for SCLC-transformed patients.

Although most transformed SCLC (tSCLC) tumors retain the same *EGFR* mutation as the primary LUAD, EGFR expression is lost upon transformation, and tumors become insensitive to EGFR-TKIs (5–7). EGFR expression in SCLC is usually lower than that in LUAD, and the mechanism of silencing or reducing EGFR expression remains unknown. tSCLCs are generally sensitive to chemotherapy agents, including platinum, etoposide, and taxanes that are used to treat conventional (de novo) SCLC (3). Inactivation of tumor suppressors *TP53* and *RB1*, a hallmark of conventional SCLC, is also ubiquitous in tSCLC. The presence of alterations in both *RB1* and *TP53* prior to EGFR-TKI treatment increases the probability that *EGFR*-mt LUAD will relapse as SCLC (8), with a 6- to 43-fold higher frequency of relapse as SCLC compared with the *EGFR*-mt LUAD population at large (9, 10).

Despite the similarities at the molecular level and in therapeutic approaches to de novo SCLC and tSCLC, the biological differences between these 2 types of SCLC remain largely underexplored. In the current study, we generate models of tSCLC directly from *EGFR*-mt patient specimens that developed clinical resistance to EGFR-TKIs. We reveal a paracrine interdependence between the neuroendocrine (NE) and non-NE components found in *EGFR*-mt tSCLC, which affects both growth and drug sensitivity. Our findings uncover distinctive biological features and a therapeutic approach for *EGFR*-mt tSCLC.

## Results

### Single-cell analyses reveal phenotypic inter- and intratumor heterogeneity in tSCLC.

**Figure 1:**
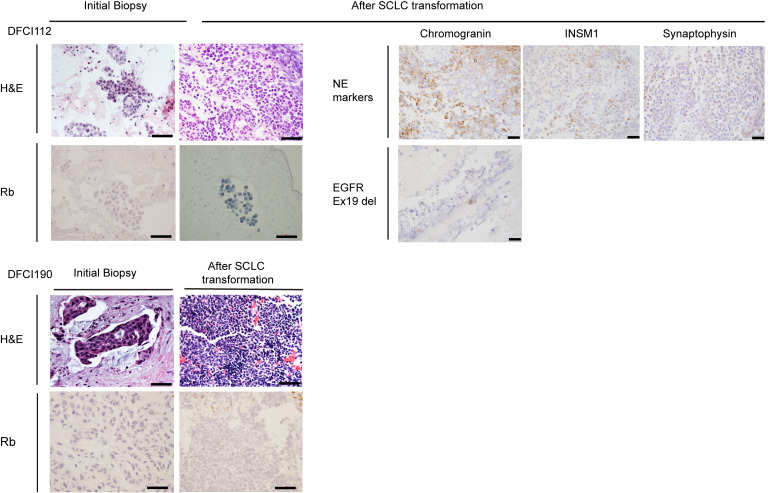
The matched pre- and posttreatment lung biopsies reveal a transformation from non-SCLC to SCLC. Hematoxylin and eosin (H&E) and IHC staining for Rb on the matched pre- and post–EGFR-TKI treatment lung biopsies of DFCI112 and DFCI190 and IHC staining for chromogranin A, INSM1, synaptophysin, and EGFR exon 19 deletion (*E746-A750del*) on post–EGFR-TKI treatment lung biopsy of DFCI112. 400×. Scale bar, 100 μm (left), 50 μm (right).

To explore the biology of tSCLC, we utilized 4 cases of *EGFR*-mt lung cancer (DFCI112, DFCI190, DFCI283, and DFCI163) with a history of SCLC transformation following EGFR-TKI treatment (Supplemental Figure 1, A–D; supplemental material available online with this article; https://doi.org/10.1172/jci.insight.197008DS1). Although initial biopsies showed typical LUAD, tumor biopsy specimens at the time of acquired resistance to EGFR-TKI demonstrated small cell morphology showing high nuclear cytoplasmic ratio and nuclear molding (Figure 1 and Supplemental Figure 2A). In DFCI112, for which tumor specimens were available for further staining, positive staining was observed for chromogranin A, INSM1, and synaptophysin, consistent with small cell carcinoma. A small subset of cells was also positively stained with EGFR E746_A750 deletion-specific antibody (Figure 1). We further obtained pleural effusion samples from these 4 cases. Xenograft models derived from these patient samples showed SCLC (DFCI112, DFCI190, and DFCI283), presenting small-sized cells with scant cytoplasm and nuclear molding (Supplemental Figure 2B). DFCI112 xenograft tumors were sensitive to combination therapy with carboplatin and etoposide consistent with the clinically observed sensitivity of tSCLC to this chemotherapy combination (3) (Supplemental Figure 2C). Unlike the other 3 models, DFCI163 xenograft tumors presented non-SCLC histology with relatively large cells with abundant cytoplasm (Supplemental Figure 2B). Although the DFCI163 individual experienced SCLC transformation earlier in the course of treatment, the malignant effusion was obtained at the time of disease progression on several SCLC-directed chemotherapies (Supplemental Figure 1D), suggesting that LUAD clones were selected as a result of selective pressure of SCLC-directed treatment. This highlights the oscillating nature of SCLC and LUAD that is common in patients with *EGFR*-mt LUAD treated with both an EGFR-TKI and chemotherapy for SCLC (5).

**Figure 2:**
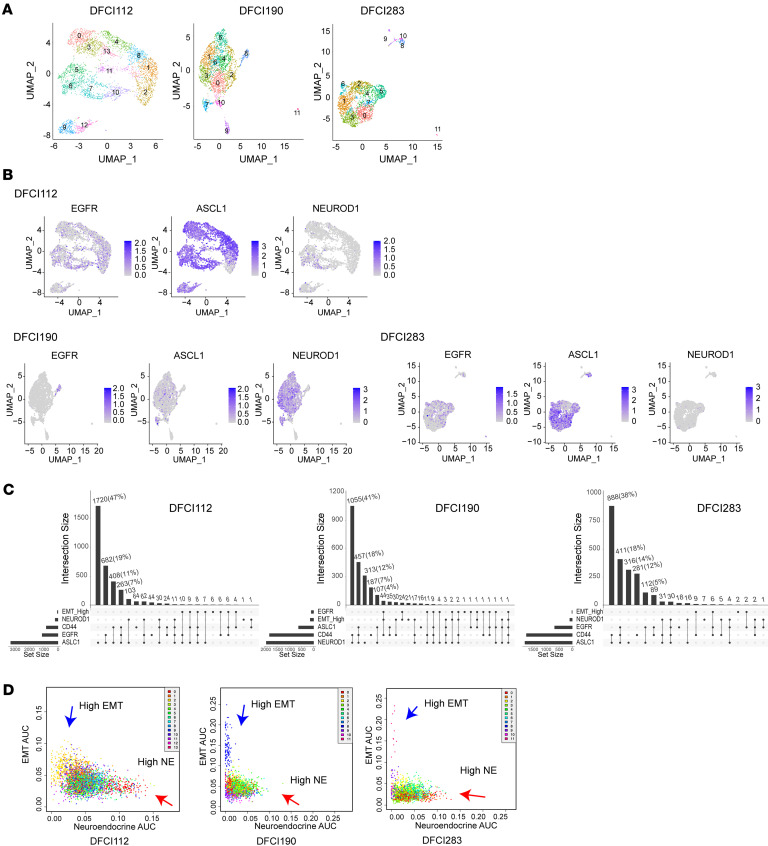
Intratumoral heterogeneity in pleural effusions of patients with *EGFR*-mt tSCLC. (**A**) UMAP representation of the scRNA-Seq data. (**B**) UMAP feature plots showing the heterogeneity of *EGFR*, *ASCL1*, and *NEUROD1*. (**C**) Upset plots showing the number of cells with unique and overlapping expression profiles of genes indicated. (**D**) The scatterplots represent the enrichment scores for NE vs. EMT signatures colored by cluster ID.

To further investigate the diversity and intratumor heterogeneity in tSCLC, we performed single-cell RNA sequencing (scRNA-Seq) on cryopreserved malignant pleural effusions with confirmed SCLC histology (DFCI112, DFCI190, and DFCI283) (Supplemental Figure 3, A–F). We conducted unsupervised clustering of the single-cell data and visualized clusters using uniform manifold approximation and projection (UMAP) representation (Figure 2A). Heatmaps of differentially expressed genes in clusters revealed intratumor heterogeneity in each sample (Supplemental Figure 3, G–I). Several recent studies have proposed to group SCLC into 4 main subtypes defined by expression of 4 transcription factors, ASCL1 (SCLC-A), NEUROD1 (SCLC-N), YAP1 (SCLC-Y), and POU2F3 (SCLC-P) (11, 12). Since little is known about the molecular subtypes in tSCLC, we investigated the expression pattern of these transcription factors in our tSCLC samples. While the majority of the cells in DFCI112 and DFCI283 expressed *ASCL1*, only a small fraction of cells was positive for *ASCL1* in DFCI190; instead, *NEUROD1*-positive cells were dominant (Figure 2, B and C, and Supplemental Figure 3J). Neither *YAP1* nor *POU2F3* was widely expressed in these samples (Supplemental Figure 3, K–M), suggesting that our tSCLC cases fall into the NE subtype (either SCLC-A or SCLC-N). We further investigated *EGFR* expression and its coexpression with *ASCL1/NEUROD1* in the samples. In DFCI112, about 32% of the cells were positive for *EGFR*, and about 96% of *EGFR*-positive cells were also positive for *ASCL1*. In DFCI190, *EGFR*-positive cells represented a minority population, and approximately 14% of *EGFR*-positive cells were also positive for *ASCL1*, whereas approximately 30% of *EGFR*-positive cells were positive for *NEUROD1*. In DFCI283, about 30% of the cells were positive for *EGFR*, and about 82% of *EGFR*-positive cells were also positive for *ASCL1* (Figure 2, B and C). Thus, tSCLC cells from primary samples revealed heterogenous gene expression of key transcription factors and *EGFR*, both inter- and intratumorally, and demonstrated distinct coexpression patterns of *EGFR* and NE marker genes.

The intratumoral heterogeneity associated with epithelial-mesenchymal transition (EMT) plays a role in resistance to chemotherapy (13) and disease progression (14–16) in de novo SCLC and other cancer types. EMT is also linked to cellular plasticity and reprogramming to an NE state in prostate cancer (17). Accordingly, we hypothesized that tSCLC may be enriched with mesenchymal gene signatures and investigated the expression of EMT markers (MSigDB: Hallmark epithelial mesenchymal transition) (18). We found high-EMT/low-NE subpopulations (blue arrows, Figure 2D) as well as a high proportion of *CD44*-positive cells in all 3 samples (22%, 85%, and 84% in DFCI112, DFCI190, and DFCI283, respectively; Figure 2C and Supplemental Figure 3, K–M). High *CD44* positivity of tSCLC samples supports the previous hypothesis that EMT/stem-like state might be involved in small cell transformation (19, 20). Further, most of the cells lacked the expression of any markers of normal fibroblasts (*ENTPD1*, *ASPN*, *FGF7*, *HTR2B*) (21) (Supplemental Figure 3N), which excludes the possibility that high-EMT/low-NE cells are benign stromal cells. These findings reveal that the primary samples of patients with tSCLC contain both populations with high NE marker expression (red arrows, Figure 2D) and a subpopulation that expresses high-EMT/low-NE markers (blue arrows, Figure 2D).

Despite the emerging subtype classification of de novo SCLC, intratumoral tumor heterogeneity is common, including focal non-NE subclusters (22). Since we identified a similar non-NE cell state in *EGFR*-mt tSCLC, we compared its transcriptomic profile with that of traditional non-NE SCLC, using RNA-Seq data generated from pure non-NE SCLC patient samples (22). Interestingly, we observed significant overlap of multiple genes related to interferon signaling and chemokines (e.g., *IFI16*, *IFITM2*, *IFITM2*, *CCL5*) as well as antigen presentation (e.g., *B2M*, *HLA-B*, *HLA-E*) (Supplemental Figure 3O). Gene set enrichment analysis further supported upregulation of antigen processing and presentation in EGFR tSCLC non-NE cells (Supplemental Figure 3P). Taken together, these data reveal that non-NE subpopulation heterogeneity of *EGFR*-mt tSCLC shares important similarities with the non-NE cell state in traditional SCLC, primarily with respect to upregulation of antigen presentation.

**Figure 3:**
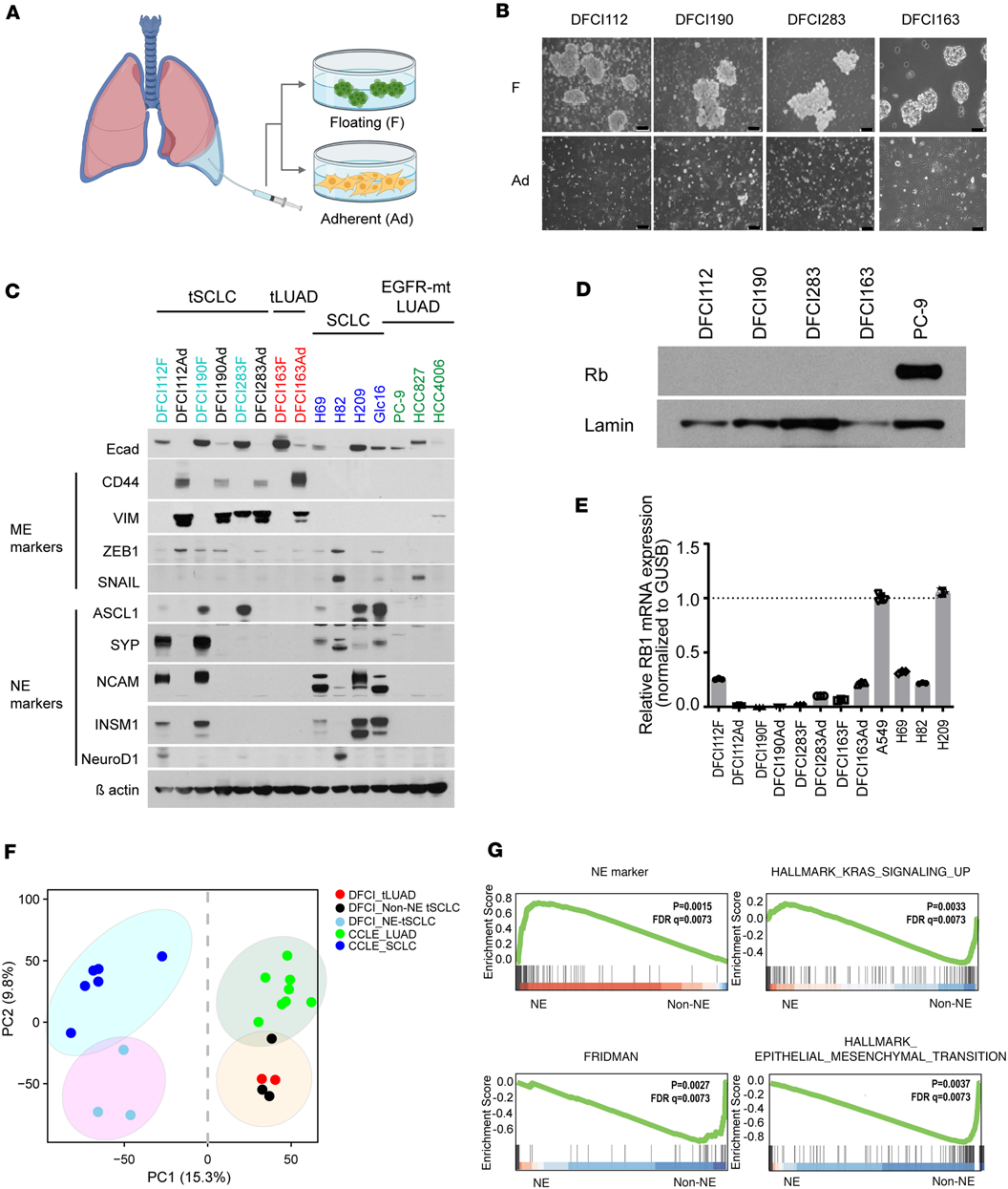
The cell lines established from SCLC-transformed patients demonstrate phenotypic heterogeneity. (**A**) The schema of cell line model creation from pleural effusions. (**B**) Phase contrast images showing the cell lines from SCLC-transformed patients growing in suspension (upper) and as monolayers (bottom). Scale bar, 50 μm. (**C**) Western blot analysis of lysates from various lung cancer cell lines as indicated. The blots were probed with antibodies against E-cadherin, mesenchymal markers (CD44, vimentin, ZEB1, and SNAIL), NE markers (ASCL1, synaptophysin, NCAM, INSM1, and NEUROD1), and β-actin (loading control). (**D**) Western blot analysis of nuclear protein extracts of various lung cancer cell lines as indicated. The blot was probed with antibodies against Rb and lamin (loading control). PC-9 is presented as a positive control. (**E**) qPCR analysis of *RB1* mRNA expression levels in various lung cancer cell lines as indicated after normalization to *GUSB* and relative to that in A549 (*n* = 3, technical replicate, mean ± SD). (**F**) Principal component analysis (PCA) of the RNA-Seq data of various lung cancer cell lines as indicated (*n* = 22). The cell lines used in this analysis are listed in Supplemental Table 6. The gene expression data of SCLC and *EGFR*-mutant LUAD cell lines was obtained from the Cancer Cell Line Encyclopedia. (**G**) Gene set enrichment analyses for NE marker, Fridman et al. (26) (senescence), KRAS signaling pathways, and EMT derived by comparing NE tSCLC cell lines with non-NE tSCLC cell lines.

Next, we successfully established 4 pairs of cancer cell lines from the pleural effusion of each patient, with one cell line consisting of floating cell clusters (DFCI112F, DFCI190F, DFCI283F, and DFCI163F) and another of adherent monolayer cells (DFCI112Ad, DFCI190Ad, DFCI283Ad, and DFCI163Ad) (Figure 3, A and B, and Supplemental Table 1). While all the adherent cell lines showed elongated spindle-like morphology, the spheroids in DFCI163F consisted of much larger cells that were reminiscent of a non-SCLC than observed in the other 3 suspension cell lines. DFCI112Ad and DFCI190Ad were capable only of limited serial propagation in vitro, and 3 monolayer cell lines (DFCI112Ad, DFCI190Ad, and DFCI283Ad) underwent senescence at later passages, as assayed by positive staining for senescence-associated β-galactosidase (Supplemental Figure 4A). To ensure that these monolayer sublines are cancer cells, we confirmed that the original *EGFR* mutations were maintained in these lines by Sanger sequencing (data not shown). H&E staining of cell pellets of the established cell lines confirmed histology consistent with the assigned diagnosis in corresponding xenograft models (Supplemental Figure 2B and Supplemental Figure 4B).

Cells growing as suspended aggregates (DFCI112F, DFCI190F, and DFCI283F) expressed variable amounts of at least one NE marker (ASCL1, synaptophysin [SYP], NCAM, INSM1, and NEUROD1), while cells with adherent morphology and DFCI163 did not express any of these markers. In accordance with their mesenchymal morphology, all adherent tSCLC sublines expressed EMT markers (CD44, vimentin, and ZEB1) (Figure 3C). Collectively, DFCI112F, DFCI190F, and DFCI283F floating subpopulations displayed an NE SCLC phenotype, while DFCI112Ad, DFCI190Ad, and DFCI283Ad adherent sublines displayed a non-NE mesenchymal SCLC phenotype, consistent with the non-NE subclusters we observed transcriptionally and the known ability to derive non-NE cell lines from traditional SCLC in vitro (23). In contrast, DFCI163F/Ad showed characteristics of non-SCLC morphologically and biologically, which was consistent with the xenograft tumor histology (Supplemental Figure 2B). Hereafter, we refer to DFCI163F/Ad as tLUAD cell lines that represent histologically confirmed non-SCLC (adenocarcinoma) cell lines established from a patient with a history of SCLC transformation, in order to distinguish from tSCLC with histological confirmation of SCLC. Based on the presence of both NE and mesenchymal cell populations in the tSCLC cell lines (DFCI112, DFCI190, and DFCI283), we concluded that the patient-derived cell lines recapitulate the phenotypic heterogeneity of the original primary samples. We also examined the tSCLC patient-derived xenograft (PDX) tumors by IHC for vimentin and CD44 (Supplemental Figure 4C). All tumors were positive for at least 1 of the 2 markers (DFCI112: positive for vimentin and CD44, DFCI190: negative for vimentin, positive for CD44, DFCI283: positive for vimentin, negative for CD44), which suggests that heterogeneity is also preserved in the PDX models.

Whole-exome sequencing (WES) of the cell lines verified that all cases harbored the same original activating *EGFR* mutation as in their diagnostic tumor specimen (Supplemental Figure 5 and Supplemental Tables 2 and 3). The only shared mutations across all tSCLC and tLUAD cell lines were in *TP53* and *EGFR* (Supplemental Figure 5 and Supplemental Table 2). *MYCL* was amplified in DFCI112, and other gene alterations common in the tSCLCs were in *PTEN* (DFCI112: 2 copies deleted; DFCI190: p. R233*) and *PIK3CA* (DFCI283: p. K111N). Using WES and targeted next-generation sequencing (OncoPanel) (24), we found deleterious mutations with concomitant loss of heterozygosity or homozygous deletion of *RB1* in DFCI112, DFCI283, and DFCI163 (Supplemental Table 2–4), while no *RB1* alterations were detected in DFCI190 either by WES or by OncoPanel. We next evaluated whether Rb expression was altered in *EGFR*-mt tSCLC tumor samples since *RB1* is invariably altered in classic SCLC (25). Tumor samples before transformation from DFCI112, DFCI190, and DFCI163 and after transformation from DFCI112 and DFCI190 were available for Rb staining. Samples after transformation were negative for Rb expression, as expected (Figure 1). Notably, pretransformed samples also lacked Rb expression (Figure 1 and Supplemental Figure 6A), consistent with a prior report (10). All established tSCLC and tLUAD cell lines exhibited loss of Rb protein expression, including DFCI190, where no genomic alterations were detected (Figure 3D and Supplemental Figure 6B). *RB1* transcript levels were confirmed to be low in all samples compared with A549 (*KRAS*^G12S^, WT-*RB1*) by qPCR (Figure 3E). These results underscore the importance of examining Rb protein expression, not only genomic sequences.

### Non-NE tSCLC is enriched with an EMT gene signature and a KRAS activation gene signature.

We further conducted transcriptomic profiling of tSCLC cell lines by bulk RNA-Seq (Supplemental Table 5) and compared the profiles with 14 cell lines (6 SCLC, 8 LUAD) with or without *EGFR* mutations from the Cancer Cell Line Encyclopedia (Supplemental Table 6 and https://sites.broadinstitute.org/ccle/). Principal component analysis (PCA) of RNA expression data revealed that the 3 NE tSCLC cell lines (DFCI112F, DFCI190F, and DFCI283F) closely resembled de novo SCLC cell lines, while non-NE tSCLC cell lines (DFCI112Ad, DFCI190Ad, and DFCI283Ad) and tLUADs (DFCI163F and DFCI163Ad) clustered together with LUAD cell lines (Figure 3F).

The first principal component (PC1) divided samples into 2 groups, NE and non-NE, while PC2 separated transformed cells and de novo cancer cells. Given the association of NE markers with floating cells and non-NE markers with adherent cells, we directly compared transcriptional phenotypes of floating and adherent cells by utilizing pathway enrichment analysis (Supplemental Figure 7A). This analysis revealed significantly enriched EMT signature (18) as well as senescence-associated gene expression signature (26) in non-NE tSCLC (Figure 3G, adjusted *P* value = 0.0073 for both signatures), consistent with our in vitro observations (Figure 3, B and C, and Supplemental Figure 4A). In addition, non-NE cells had an upregulated *KRAS* signaling gene set along with inflammatory response gene sets (Figure 3G and Supplemental Figure 7A), in keeping with the previous observation that EMT in SCLC can be driven by activated KRAS signaling and our scRNA-Seq analyses (Supplemental Figure 3, O and P) (15). The accelerated cellular senescence phenotype in non-NE tSCLC agrees with the previous study showing that activation of MAPK, which is one of the downstream cascades of RAS, induces cell cycle arrest and senescence in SCLC subtypes (27). We further examined expression of specific ERK transcriptional target genes (*DUSP4*, *ETV5*, *ETV4*, *DUSP6*, *SPRY2*, *PHLDA1*) (28) and found that *DUSP6*, *SPRY2*, and *PHLDA1* were upregulated in non-NE tSCLC (Supplemental Figure 7B). To validate these results in the original patient samples, we examined the enrichment for the KRAS hallmark pathway gene set (MSigDB: Hallmark KRAS signaling up) (18) in scRNA-Seq data and identified high-KRAS/low-NE subpopulations in DFCI190 and DFCI283 (green arrows, Supplemental Figure 7C). Furthermore, PC2 could effectively distinguish between transformed and de novo SCLC cells, which suggests that transformed cells might share some transcriptomic signatures that make the cells prone to transformation. Finally, comparison between NE tSCLC and de novo SCLC (Supplemental Figure 7D) and between non-NE tSCLC and de novo SCLC (Supplemental Figure 7E) revealed that the Hallmark-EMT pathway was the top enriched pathway in both NE and non-NE tSCLC. In addition, ERK and Hallmark-KRAS activation pathways as well as senescence-associated gene expression signature (Fridman et al., ref. 26) were enriched in non-NE tSCLC relative to de novo SCLC (Supplemental Figure 7E). To further validate this observation, we calculated the transcriptional MAPK Pathway Activity Score (29). Non-NE tSCLC showed relatively higher transcriptional ERK activity score compared with de novo SCLC (Supplemental Figure 7F). These findings further support that tSCLC is characterized by the EMT phenotype, and the ERK pathway is activated in non-NE tSCLC, which is likely involved in acquisition of the senescence feature similar to the previous study (27).

### Coculturing with adherent counterparts increases proliferation of EGFR-mt NE tSCLC cells.

**Figure 4:**
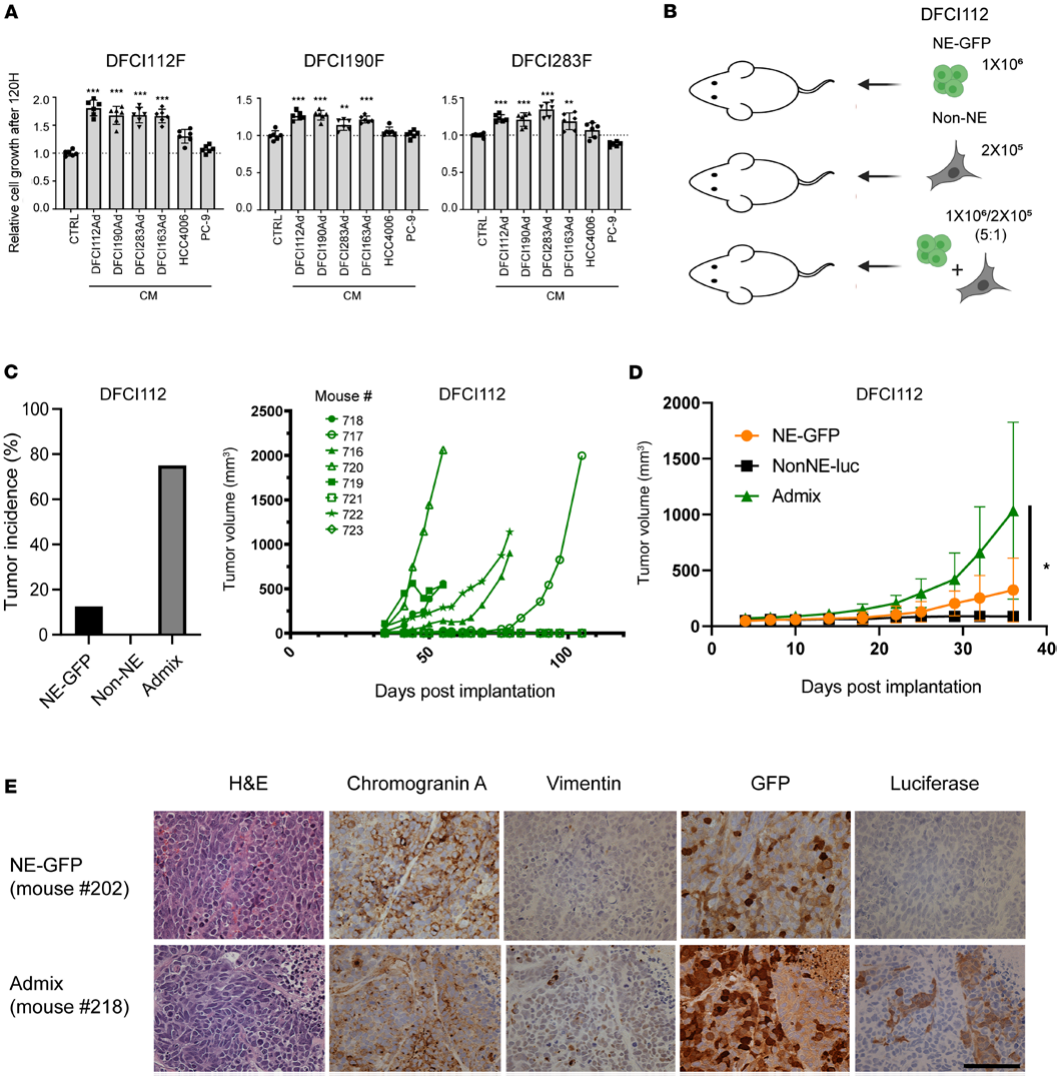
Crosstalk between NE and non-NE tSCLC cells promotes growth of NE cells and supports successful engraftment. (**A**) Effects of CM from various indicated lung cancer cell lines with adherent morphology on growth of the NE tSCLC cells. Data represent mean ± SD (*n* = 6, technical replicates). **= *P* ≤ 0.01, ***= *P* ≤ 0.001 by 1-way ANOVA with Dunnett’s multiple comparisons test. (**B**) Schematic representation of the in vivo experimental design. (**C**) Left: The tumor engraftment rate in nude mice. No tumors were observed in mice injected with pure non-NE population. Right: Tumor volume measurement of transplanted tumors after injection of pure EGFP-expressing DFCI112F cells, unlabeled DFCI112Ad cells, or admixed cells into nude mice. Data are presented as mean ± SD (8 mice per condition). (**D**) Tumor volume measurement of transplanted tumors after injection of pure EGFP-expressing DFCI112F cells, luciferase-labeled DFCI112Ad cells, or admixed cells into NSG mice. Data are presented as mean ± SD (8 mice per condition). *= *P* ≤ 0.05 by 1-way ANOVA with Turkey’s multiple comparisons test. (**E**) H&E and IHC image (400×) for chromogranin A, vimentin, GFP, and luciferase of PDX tumors from admixed cells (experiment from **D**, mouse number 202 and 218). Scale bar, 100 μm.

SCLC cells derived from genetically engineered mouse models have been reported to be composed of NE and mesenchymal non-NE cells, and crosstalk between the cells strongly influences their behavior (15). To investigate this crosstalk in human tSCLC models, we tested the effect of conditioned media (CM) from adherent non-NE tSCLC cell lines on NE tSCLC lines. CM from the corresponding non-NE tSCLC cells significantly enhanced the proliferation of their respective NE tSCLC counterparts (Figure 4A). Furthermore, CM from other non-NE tSCLC also significantly enhanced the proliferation of other NE tSCLC cells. In contrast, CM from *EGFR*-mt LUAD cell lines (HCC4006 and PC-9) did not enhance the proliferation of the NE tSCLCs (Figure 4A), suggesting that the factors specifically secreted by non-NE tSCLC cells support the growth of NE tSCLC cells.

### EGFR-mt NE tSCLC cells (DFCI112F) show enhanced engraftment in both nude and NOD/SCID IL-2R-γ–null mice when admixed with their non-NE counterparts (DFCI112Ad).

To further investigate crosstalk between NE and non-NE cells in vivo, GFP-labeled NE tSCLC cells (DFCI112F) and unlabeled non-NE cells (DFCI112Ad) were implanted subcutaneously into nude mice either as pure populations or admixed at a 5:1 ratio (Figure 4B). We used this ratio based on pilot in vivo experiments establishing the optimal growth of NE cells (data not shown). Cell engraftment rate in the admixed group was notably higher than in the NE-GFP or non-NE group (6/8 [75%], 1/8 [12.5%], 0/8 [0%], respectively) (Figure 4C). To ensure that this is not merely due to the selection of cells that can survive in the nude mice immune system, we repeated the same experiment using NOD/SCID IL-2R-γ–null (NSG) mice, which are one of the most highly immunodeficient strains. Non-NE cells were labeled with luciferase for this experiment. Similar to the prior experiment using the nude mice, significantly superior engraftment was observed in the admixed group (Figure 4D). We did not detect any metastatic lesions either in lungs or in livers of engrafted mice (data not shown). In the admixed tumors, we could identify the luciferase-positive cells (Figure 4E). These cells were negatively stained with chromogranin A, while staining for vimentin, which is exclusively expressed in non-NE cells in vitro, was only partially positive in vivo (Figure 4E), which raises the possibility that the EMT marker expression profile of non-NE cells may be different between in vitro and in vivo because of cell plasticity. In vitro culture, which is performed under a controlled environment, may select for cells with more extreme mesenchymal characteristics. In vivo tumors derived from in vitro–cultured non-NE cells may lose some of the mesenchymal marker expression while maintaining their non-NE phenotype. Nevertheless, these results suggest that coimplantation of NE and non-NE cells supported the engraftment and in vivo expansion of tSCLCs.

### EGFR expression in the EGFR-mt NE tSCLC cell line (DFCI112F) is regulated by EZH2.

**Figure 5:**
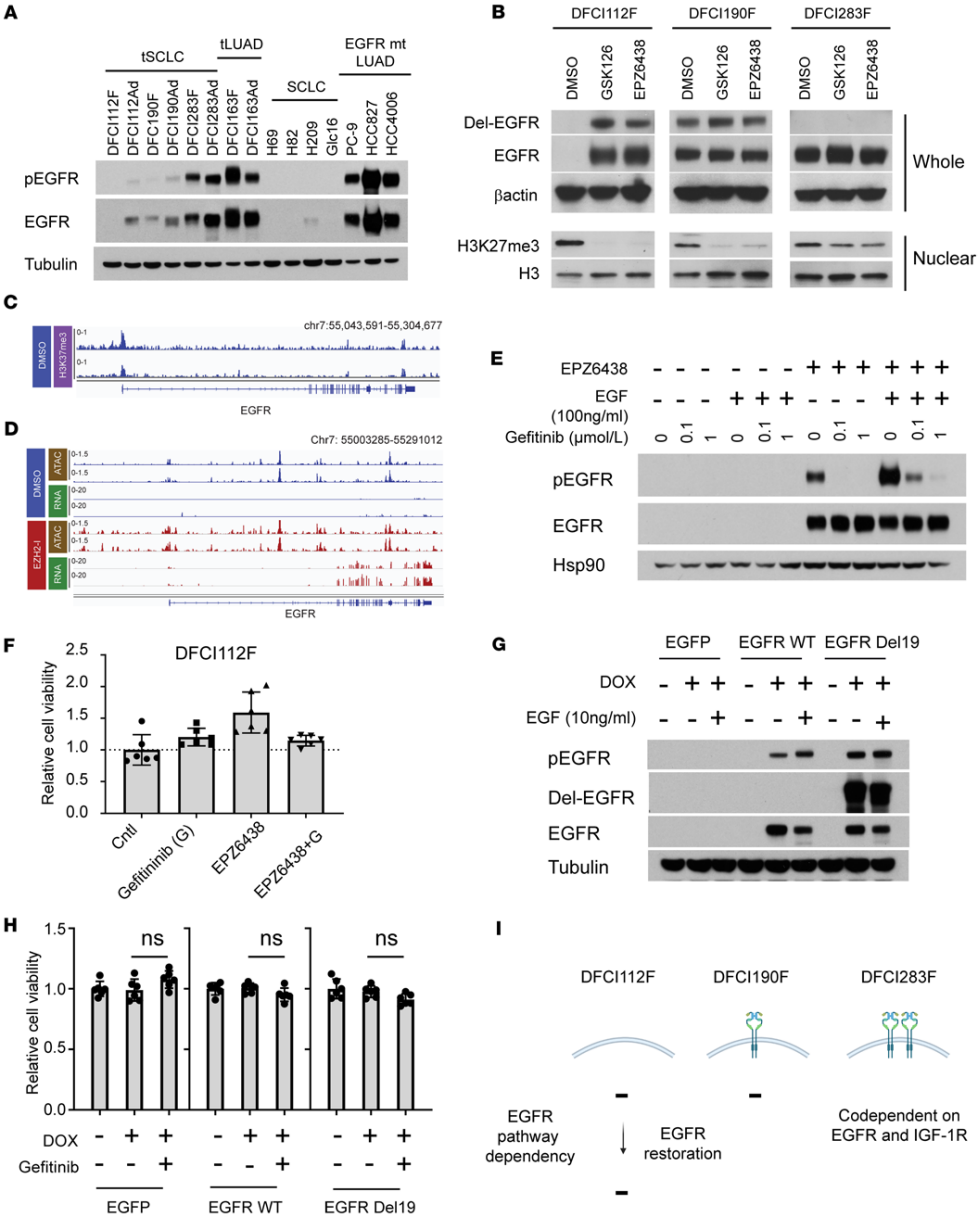
Neither endogenous nor exogenous EGFR expression sensitizes DFCI112F cells to EGFR inhibition. (**A**) Western blot analysis of lysates from indicated cell lines. Blots were probed for phospho-EGFR (Tyr1068), EGFR, and tubulin (loading control). (**B**) Western blot analysis of NE-tSCLC cells pretreated with DMSO or 10 μmol/L of GSK126 or EPZ6438 for 10 days. Blots were probed for Del-EGFR, β-actin (loading control), H3K27me3, and H3. *Del EGFR antibody (*E746-A750del* specific, CST) used for this assay did not recognize Del-EGFR protein in 283F (*p*. *L747_A750delinsP*). Whole, whole-cell extracts; Nuclear: nuclear extracts. (**C**) H3K27me3 ChIP-Seq signal traces in EGFR locus in DFCI112F. (**D**) RNA-Seq and ATAC-Seq signal traces at the EGFR locus after DMSO or EZP6438 (5 µmol/L, 9 days). (**E**) Western blot analysis of DFCI112F. The cells were pretreated either with DMSO or with 10 μmol/L of EPZ6438 for 10 days and subsequently treated either with DMSO or gefitinib as indicated ± EGF (100 ng/mL, 15 minutes). Blots were probed for phospho-EGFR (Tyr1068), EGFR, and Hsp90 (loading control). (**F**) Relative viability of DFCI112F cells pretreated with DMSO or EPZ6438 (10 µmol/L, 10 days), then treated with DMSO, gefitinib (1 µmol/L), EPZ6238 (10 µmol/L), or their combination. The cell viability was assessed by CellTiter-Glo (CTG) after 120 hours (*n* = 6, technical replicates, mean ± SD). (**G**) Western blot analysis of DFCI112F infected with DOX-inducible EGFP, DOX-inducible exon 19 del-EGFR or DOX-inducible WT-EGFR expressing lentiviruses and grown in the presence or absence of DOX with or without acute EGF stimulation (10 ng/mL for 15 minutes). Blots were probed for phospho-EGFR (Tyr1068), Del-EGFR, total EGFR, and tubulin (loading control). (**H**) Relative cell viability of DFCI112F infected as described in **G**. The cells were grown ±DOX and treated with DMSO or 1 μmol/L of gefitinib. The cell viability was assessed by CTG after 120 hours (*n* = 6, technical replicates, mean ± SD). (**I**) The schema illustrating EGFR pathway dependency in NE tSCLC cells.

tSCLC with *EGFR*-activating mutations is widely assumed to be resistant to EGFR pathway inhibition because of lack of EGFR expression. To validate this assumption, we evaluated EGFR protein expression in *EGFR-*mt tSCLC cell lines and found that they exhibited great variation in expression level. DFCI112F lacked EGFR expression, whereas DFCI190F and DFCI283F retained some level of expression (DFCI283F > DFCI190F) (Figure 5A). These results were verified with qPCR (Supplemental Figure 8A). Non-NE tSCLC cell lines demonstrated higher EGFR expression than NE tSCLC cell lines, including DFCI112Ad, whose NE counterpart had no EGFR expression (Figure 5A). This heterogeneity in EGFR expression in NE tSCLC cells prompted us to test cellular differences in sensitivity to EGFR-TKI. Consistent with the clinical resistance of tSCLC to EGFR-TKIs, both NE and non-NE tSCLC were largely resistant to osimertinib (IC_50_ > 1 μmol/L) (Supplemental Figure 8B), although DFCI283F retained some level of sensitivity. siRNA-mediated knockdown of EGFR in DFCI283F reduced cell viability to 53% (Supplemental Figure 8C). To explore the possibility that activation of alternative receptor tyrosine kinases (RTKs) mediates resistance to the EGFR pathway in tSCLC cells, we analyzed both NE and non-NE tSCLC cell lines using human phosphorylated RTK (p-RTK) arrays (Supplemental Figure 8D). Three RTKs (EGFR, insulin R, and IGF1R) were found to be phosphorylated in NE tSCLC, whereas 6 RTKs (EGFR, PDGFRβ, AXL, ERBB2, insulin R, and IGF1R) were phosphorylated in non-NE tSCLC. Accordingly, we tested the combination of OSI906 (an IGF1R/insulin R inhibitor) with osimertinib in NE tSCLC and sunitinib (a multikinase inhibitor including PDGFRβ), R428 (an AXL kinase inhibitor), and OSI906 with osimertinib in non-NE tSCLC (Supplemental Figure 8E). The combination of OSI906 and osimertinib drastically reduced the cell viability in both DFCI283F and DFCI283Ad, which suggests that DFCI283 cells are codependent on EGFR and IGF1R signaling pathway.

Classic SCLC is reported to have lower EGFR expression than LUAD (30, 31), yet how EGFR expression is regulated in *EGFR*-mt tSCLC remains unknown. The level and pattern of *EGFR* expression differed between primary samples and cell lines. In DFCI112, single-cell analysis of original effusion samples revealed most *EGFR*-positive cells expressed *ASCL1*, while *ASCL1*-positive NE cells in the cell line showed no expression of *EGFR*, suggesting epigenetic regulation and/or selection pressure in the culture condition may underlie this discrepancy. EZH2, a histone methyltransferase and the catalytic subunit of the polycomb repressive complex 2, regulates androgen receptor (AR) expression in advanced prostate cancer, and EZH2 inhibition restores sensitivity to antiandrogen therapy (32). We examined whether this epigenetic regulation of AR also applied to regulation of EGFR expression in *EGFR*-mt tSCLC. When treated with the EZH2 inhibitors GSK126 and EPZ6438, endogenous mutant EGFR expression was restored uniquely in DFCI112F (Figure 5B and Supplemental Figure 8F). The siRNA-mediated knockdown of EZH2 in DFCI112F increased *EGFR* mRNA expression (Supplemental Figure 8, G and H). To examine if this EZH2-mediated EGFR regulation also occurred in *EGFR*-WT SCLC, we treated classic SCLC cell lines (H69, H209, and H345) with the EZH2 inhibitor GSK126 (Supplemental Figure 8, I and J). All 3 cell lines demonstrated some level of EGFR restoration, suggesting EZH2-mediated silencing of EGFR is not limited to *EGFR*-mt tSCLC but extends to de novo SCLC. However, the unique impact of EZH2 inhibition on EGFR expression suggested that higher levels of regulation could be at play in DFCI112F. Consistent with these observations, chromatin immunoprecipitation sequencing (ChIP-Seq) confirmed histone H3 trimethylated at lysine 27 (H3K27me3) enrichment at the promoter region of *EGFR* (Figure 5C) in DFCI112F. RNA-Seq and an assay for transposase-accessible chromatin combined with next-generation sequencing (ATAC-Seq) further verified increased transcription of *EGFR* as well as chromatin accessibility at the promoter region of *EGFR* upon EZH2 inhibitor treatment (Figure 5D).

Next, we investigated whether restoration of mutant *EGFR* expression is sufficient to resensitize DFCI112F to EGFR inhibitor treatment. Although EZH2 inhibition functionally restored EGFR stimulation by EGF, and EGFR phosphorylation was inhibited by gefitinib (Figure 5E), the cells did not become sensitive to gefitinib (Figure 5F). We further tested whether exogenous expression of either EGFR-WT or EGFR-mt resensitizes DFCI112F to EGFR-TKI using a doxycycline-inducible system (Figure 5G). Neither WT nor exon 19 del-EGFR exogenous expression restored sensitivity to gefitinib in DFCI112F (Figure 5H). This finding indicates that both endogenous and exogenous EGFR expression failed to rewire the signaling pathway dependencies to EGFR in DFCI112F cells. Thus, loss of EGFR expression alone cannot explain the lack of response to EGFR inhibition in *EGFR*-mt tSCLC (Figure 5I).

### Non-NE tSCLC cells (DFCI112Ad, DFCI190Ad, and DFCI283Ad) are sensitive to MEK inhibition.

**Figure 6:**
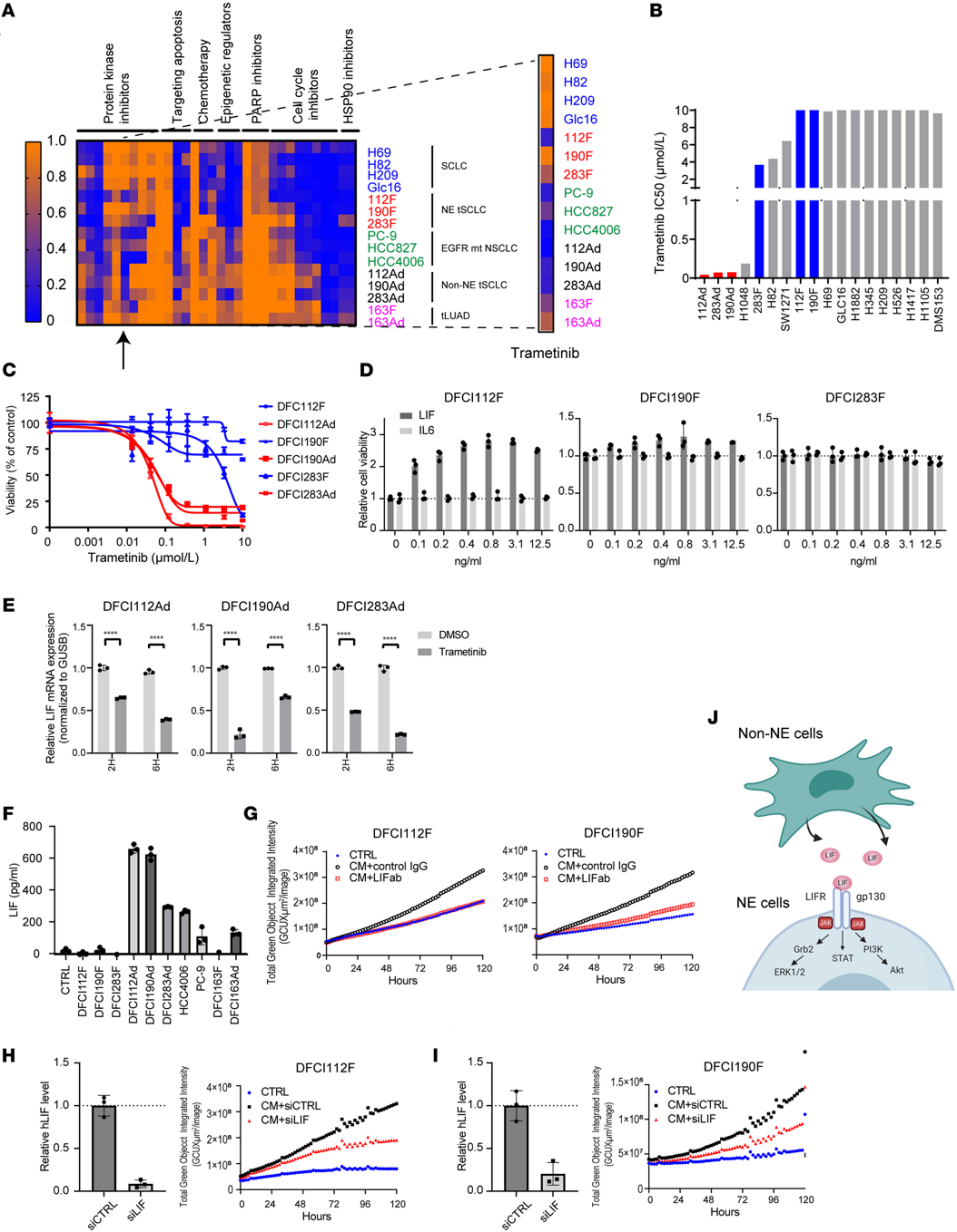
The non-NE tSCLC cells (DFCI112Ad, DFCI190Ad, and DFCI283Ad) are vulnerable to MEK inhibition. (**A**) Heatmap of cell viability after 5 days’ treatment with 1 μmol/L of each single agent (rows) per various lung cancer cell lines (columns) relative to DMSO control. Commercially available agents used are in Supplemental Table 6. (**B**) Bar graph of IC_50_ values of various indicated lung cancer cell lines in response to trametinib. IC_50_ of NE and non-NE tSCLC cell lines is shown in red and blue, respectively. (**C**) Dose-response curve of trametinib in tSCLC cell lines (blue: NE, red: non-NE). Cell viability was assessed by CTG after 120 hours (*n* = 6, technical replicate, mean ± SD). (**D**) NE tSCLC cell viability was assayed by CTG following treatment with indicated concentration of LIF and IL-6 for 5 days. (**E**) qPCR analysis of *LIF* mRNA expression levels in non-NE tSCLC cell lines treated with 10 nmol/L trametinib 2 and 6 hours. Gene expression was normalized to *GUSB* and shown as relative to that of DMSO-treated control (*n* = 3, technical replicate, mean ± SD). ****= *P* ≤ 0.001 by 2-way ANOVA with Šídák’s multiple comparisons test. (**F**) Various cell lines were analyzed for LIF expression by ELISA. Data are presented as mean ± SD (*n* = 3. Technical replicates). (**G**) GFP-expressing DFCI112F and DFCI190F cells were incubated with CM from their corresponding adherent cell line pairs pretreated with either LIF neutralizing antibody (500 ng/mL) or control IgG and monitored in real time via IncuCyte imager for green fluorescence intensity. (**H** and **I**) GFP expressing DFCI112F (**H**) and DFCI190 (**I**) cells were incubated with CM from their adherent cell line pairs pretreated with siRNA against LIF or unrelated sequence and monitored via IncuCyte imager. LIF knockdown efficiency was tested using ELISA (*n* = 3, technical replicate, mean ± SD). (**J**) The schema illustrating the interaction between NE and non-NE tSCLC cells through LIF.

Since EGFR inhibition had little impact even when its expression was restored, we looked for alternative therapeutic vulnerabilities. Since current treatment strategies for tSCLC consist of chemotherapy and mainly target NE cells, we were especially interested in identifying selective drug vulnerabilities of non-NE cells, hoping not only to target both NE and non-NE cells but also to target the crosstalk between them. We performed drug testing using 32 commercially available compounds at 2 concentrations (100 nmol/L and 1 μmol/L) on NE and non-NE tSCLC cell lines separately (Supplemental Table 6). Cell viability was measured after 120 hours of drug exposure (Figure 6A and Supplemental Figure 9A). Notably, non-NE tSCLC cells exhibited higher sensitivity to the MEK inhibitor trametinib (IC_50_ < 80 nmol/L), compared with their NE tSCLC counterparts (Figure 6, B and C), which was accompanied by poly (ADP-ribose) polymerase (PARP) cleavage, following exposure to 0.1 and 1.0 μmol/L of trametinib (Supplemental Figure 9B). These findings are consistent with the RNA-Seq data demonstrating high expression of *DUSP6*, a negative regulator of ERK1/2 signaling that correlates with trametinib sensitivity (33), and enrichment of a KRAS signaling signature in non-NE cells (Figure 3G and Supplemental Figure 7, A–C, E, and F). We also tested sensitivity to PI3K/mTOR inhibitors (Supplemental Figure 9, C and D). While NE cells showed sensitivity to PI3K inhibitors, non-NE cells had 2 to 8 times higher IC_50_, suggesting that the cells switch pathway dependency from PI3K to MEK upon acquisition of a mesenchymal phenotype.

### MEK inhibition targets the crosstalk between NE and non-NE tSCLC cells.

To further investigate the effect of MEK inhibition on the crosstalk between NE and non-NE cells, we searched for the growth factors/cytokines secreted by non-NE cells and responsible for mediating the growth of NE cells (Figure 4A). First, we analyzed the CM of non-NE cells using a custom multiplex ELISA (Supplemental Figure 10A). We confirmed that supernatants of pure non-NE cells demonstrated high levels of inflammatory cytokines CCL5, IL-6, IL-8, CCL2, CXCL10, and IL-10. Among them, IL-6 and IL-8 are implicated in promoting growth of lung cancer (34, 35), but neither affected the growth of *EGFR*-mt NE SCLC cells (Figure 6D and data not shown). We further evaluated bulk RNA-Seq data for evidence of cytokines expressed, those not part of the ELISA, specifically in non-NE tSCLC cells and observed selective upregulation of leukemia inhibitory factor (LIF) (Supplemental Figure 10B). Moreover, short-term exposure (2 hours, 6 hours) of non-NE cells to 10 nmol/L of trametinib significantly reduced *LIF* mRNA expression (Figure 6E, *P* ≤ 0.0001). A multifunctional cytokine of the IL-6 cytokine family (36), LIF plays an important role in maintaining pluripotency of embryonic stem cells and induced pluripotent stem cells (37). LIF also elicits proliferation of various cancers (38) and is involved in chemotherapy resistance (39). ELISA verified that all 3 non-NE tSCLCs secreted high levels of LIF in the CM (292–657 pg/mL) (Figure 6F), though exogenous LIF administration enhanced the growth of DFCI112F and DFCI190F but not DFCI283F (Figure 6D). Pretreating CM with a neutralizing antibody against LIF prevented CM-induced cell proliferation in DFCI112F and DFCI190F (Figure 6G). To further ensure that LIF secreted from non-NE tSCLC mediates the growth of NE tSCLC, we assessed the effect of conditioned media from LIF siRNA-treated non-NE tSCLC cells on NE tSCLC cells. As expected, depletion of LIF from CM prevented cell proliferation of DFCI112F and DFCI190F (Figure 6, H and I). Collectively, these results demonstrate that non-NE tSCLC cells regulate growth of NE counterparts at least partially by secreting LIF (Figure 6J) and that trametinib, in addition to having direct effects on the non-NE cells, may also block cytokine-mediated crosstalk between NE and non-NE cells.

### MEK inhibitor combinations target intratumor heterogeneity in EGFR-mt tSCLC.

**Figure 7:**
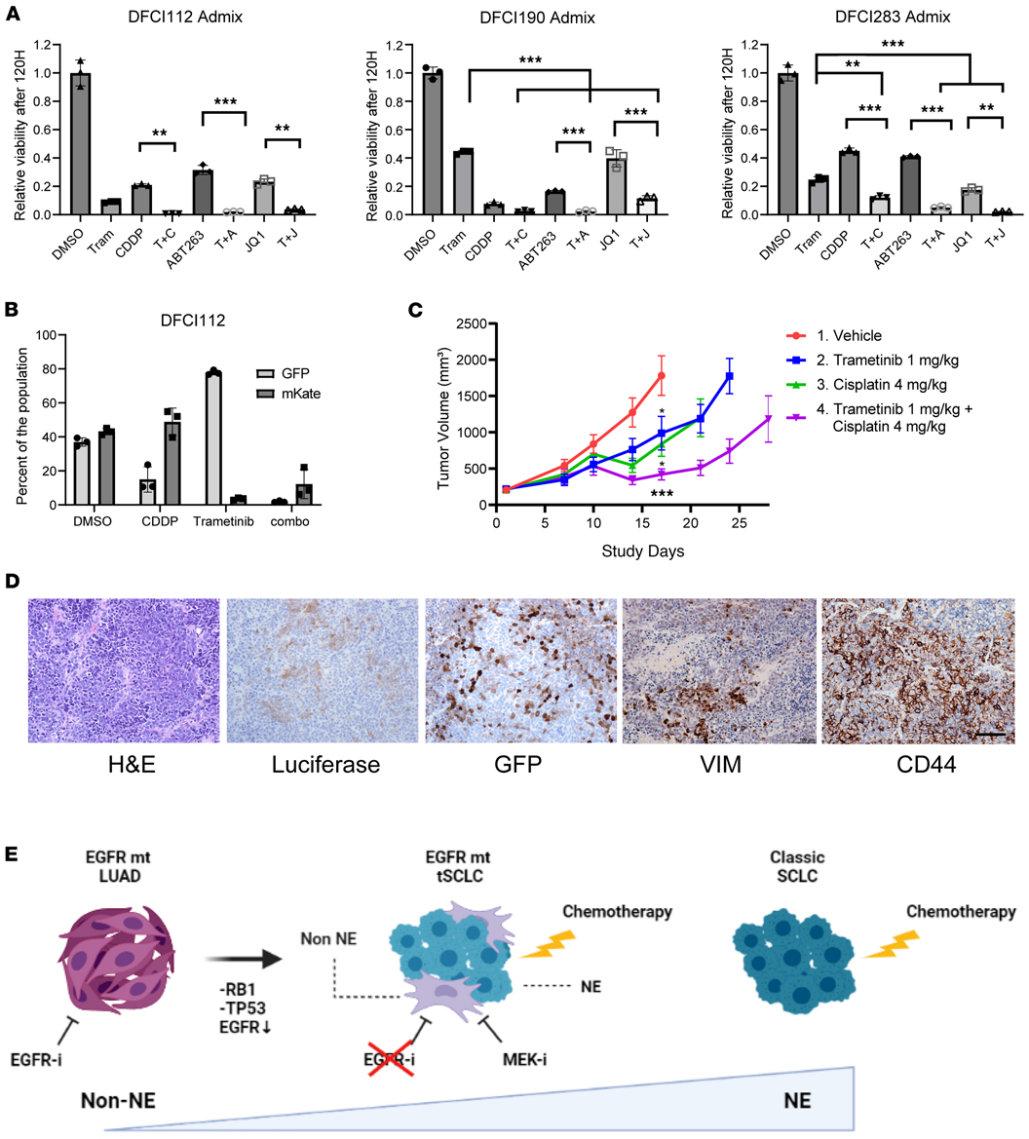
MEK inhibitor combination with cisplatin effectively targets intratumor heterogeneity in tSCLC cells. (**A**) The NE cells and the non-NE cells were combined at a ratio of 1:1, and the cell viability after indicated treatments for 5 days was assayed by CTG (*n* = 3, technical replicate, mean ± SD). Treatment concentration: trametinib (T) 0.6 μmol/L, cisplatin (C) 10 μmol/L, T+C 0.6/10 μmol/L, ABT263 (A) 0.6 μmol/L, T+A 0.6/0.6 μmol/L, JQ-1 0.6 μmol/L. **= *P* ≤ 0.01, ***= *P* ≤ 0.001 by 1-way ANOVA with Turkey’s multiple comparisons test. (**B**) Flow analysis of GFP-expressing DFCI112F cells and mKate-expressing DFCI112Ad cells combined at 1:3 ratio and treated with cisplatin or trametinib alone or in combination. After 120 hours of treatment, the viable cell populations were analyzed (*n* = 3, technical replicate, mean ± SD). (**C**) Trametinib plus cisplatin treatment showed superior efficacy over single-agent treatment in the NE-GFP/non-NE-luciferase (4:1) admixed xenografts. *= *P* ≤ 0.05, ***= *P* ≤ 0.001 by 1-way ANOVA with Turkey’s multiple comparisons test. (**D**) H&E and IHC staining for luciferase, GFP, vimentin, and CD44 on vehicle-treated xenograft tumors. 200×. Scale bar, 100 μm. (**E**) Schematic of intratumor heterogeneity in tSCLC targetable with combined chemotherapy and MEK inhibitors.

The current standard-of-care treatment for extensive-stage SCLC is platinum-based chemotherapy with immune checkpoint inhibition (40, 41). Based on the unique sensitivity of the non-NE tSCLC cells to MEK inhibition, we hypothesized that adding trametinib to a treatment currently used for de novo SCLC could increase the antitumor effect specifically in *EGFR*-mt tSCLC. This approach would not only target both non-NE and NE tSCLC populations but also impact their crosstalk. As navitoclax and JQ-1 both have an antitumor efficacy on SCLC models (42, 43), we selected these 2 compounds along with cisplatin from the 32 drugs used for screening (Figure 6A) to test the combination effect with trametinib in vitro. NE and non-NE cells were combined in a ratio of 1:1, and cell viability following cisplatin, navitoclax, or JQ-1 alone or in combination with trametinib was assessed. The trametinib combinations were significantly more effective than the single-agent treatments (*P* ≤ 0.01) (Figure 7A). To further verify the selective sensitivity of non-NE cells to trametinib, we combined GFP-expressing DFCI112F cells and mKate-expressing DFCI112Ad cells at a 1:3 ratio and treated with cisplatin or trametinib alone or in combination. The GFP and mKate positivity was assessed by flow cytometry. DFCI112Ad-mKate cells showed selective sensitivity to trametinib (DFCI112F-GFP versus Ad-mKate: 77.8% vs. 3.5%), and the combination was effective in both DFCI112F-GFP cells and Ad-mKate cells (Figure 7B).

Next, we evaluated the efficacy of combination therapy with trametinib and cisplatin in vivo using the DFCI112 model. We labeled non-NE cells with luciferase and implanted them together with GFP-labeled NE cells at a 4:1 (NE/non-NE) ratio in NRG (NOD/Rag^1/2null^/IL-2Rgamma^–/–^) mice. Cisplatin plus trametinib treatment significantly slowed tumor growth compared with treatment with cisplatin or trametinib alone (*P* ≤ 0.001) (Figure 7C). Trametinib and cisplatin monotherapy showed similar efficacy. Immunohistochemical analyses showed the existence of both GFP-positive and luciferase-positive cells in vehicle-treated tumors (Figure 7D). The majority of cells were positive for GFP, and luciferase-positive cells were less frequent, consistent with prior in vivo findings (Figure 4E).

## Discussion

A lineage switch from *EGFR*-mt LUAD to SCLC is an established mechanism of resistance to EGFR-TKIs and is currently treated the same as de novo SCLC. Yet, important questions remain of whether the biology of tSCLC differs from that of de novo SCLC and, more importantly, whether tSCLC should be treated differently from de novo SCLC. tSCLC cases resemble classic SCLC on many levels, including the ubiquitous nature of *TP53* and *RB1* loss, drug sensitivity, and gene expression profiles (4, 5). Tumors are generally sensitive to platinum-based chemotherapy (1, 3). Further, the clinical outcomes of tSCLC mimic classic SCLC, with a rapid disease course and transient response to SCLC-directed chemotherapies (9). Here, we demonstrated that *EGFR*-mt tSCLC is composed of both NE cells and mesenchymal-like non-NE cells. These 2 populations of cells existed in the original patient specimens from individuals who had clinically undergone SCLC transformation following EGFR-TKI therapy. While tumor heterogeneity is increasingly acknowledged to be an important feature of malignant tumors, a major challenge lies in assessing its role in tSCLC, because of paucity of study materials; a single site biopsy often obtained for diagnostic purposes is not representative of the entire tumor, and surgical resection of progressed lesions under therapy is uncommon. In this study, further functional studies on tumor diversity in tSCLC were made possible by generating models directly from patient specimens. While a heterogeneous phenotype of original tumors consisting of NE and non-NE cancer cells was successfully recapitulated in cell line models, we also detected some discrepancies in gene and protein expression profiles between the primary samples and corresponding cell lines. This could be due to the selection of preexisting subclones or phenotypic changes occurring during cell line creation and culturing. Those issues point out some limitations of cell lines as preclinical models, necessitating careful interpretation of observations, especially when studying cancers with highly plastic and diverse characteristics.

Prior studies, using a genetically engineered mouse model of SCLC, have demonstrated that both NE and non-NE populations can exist within the same tumor and arise from a common origin (15, 16, 44). The non-NE cells when admixed with the NE cells led to enhancement in the development of metastases. The transition from an NE cell to a non-NE cell could be achieved through various means, such as ectopic expression of *Ras*^V12^ (15), MYC (44, 45) or activation of Notch (16). *Ras*-mediated acquisition of non-NE phenotype is particularly intriguing given that we found that non-NE tSCLC cells were enriched for KRAS signaling pathways (genes transcriptionally upregulated by activated RAS) and EMT hallmark genes compared with NE tSCLC cells. In addition, they were sensitive to MEK inhibition. Our previous study also demonstrated that introduction of *NRAS*^Q61K^ leads to the acquisition of mesenchymal characteristics, and *NRAS*^Q61K^-transduced mesenchymal SCLC shows marked sensitivity to MEK inhibition (46).

Although intratumoral heterogeneity within human SCLC tumors has been reported, including the presence of non-NE cells with EMT features that are enriched following platinum-based chemotherapy, increasing evidence suggests that tSCLC is more enriched with genes involved in stemness and EMT than de novo SCLC (19, 20). Since activation of the RAS pathway, which locates downstream of EGFR, can lead to acquisition of mesenchymal phenotype in SCLC, it is plausible that EGFR pathway activation in *EGFR*-mt tSCLC may be involved in establishing heterogeneous tumor populations that include mesenchymal cells through RAS-MEK-ERK cascade activation. Indeed, recent evidence supports this hypothesis and demonstrated that ERK-CBP/p300-ETS axis promotes a shift between NE and non-NE phenotypes by expressing mutant KRAS and EGFR in SCLC cell lines (47). Furthermore, similar to a recent study (22), we did not observe upregulation of YAP in non-NE tSCLC, supporting a potential direct role for RAS signaling downstream in promoting this phenotype. Yet it is also known that other factors such as AXL and REST can promote non-NE differentiation in SCLC, and thus the unique mechanisms that can reinforce this shift in tSCLC relative to SCLC will be important areas of future study.

How to efficiently target intratumor heterogeneity and the biological interaction between the different subpopulations in tSCLC models, as demonstrated in the current study, has not previously been described to our knowledge. The interaction between the NE and non-NE cells and the sensitivity of the non-NE cells to MEK inhibition suggest that therapeutic strategies for *EGFR*-mt tSCLC may need to be different than for conventional SCLC. While the NE *EGFR*-mt tSCLCs are sensitive to agents used to treat de novo SCLC (Figure 6A), MEK inhibition significantly enhances the treatment efficacy in vitro and in vivo of the admixed NE and non-NE *EGFR*-mt tSCLC. Whether this approach could be studied clinically in *EGFR*-mt tSCLC given the known toxicities of MEK inhibition remains to be determined. Moreover, although our studies identified an important role for LIF in mediating crosstalk between non-NE and NE subpopulations, it is likely that additional secreted factors are involved, and identifying targets that inhibit cytokine secretion more broadly upstream is likely to be more impactful than neutralizing any one factor. As such, additional approaches, including maintenance MEK inhibitor therapy or identifying additional kinase targets, may need to be identified. However, our studies do highlight the need for and effect of targeting the non-NE population of cells in *EGFR*-mt tSCLC.

The comparison of the upregulated genes in the non-NE tSCLC cluster with those in MHC I^hi^ non-NE de novo SCLC cases revealed not only the differences but also some overlapped signatures including genes related to antigen presentation and interferon signaling (Supplemental Figure 3, O and P). Our previous work demonstrated that non-NE SCLC uniquely upregulates MHC I and displays intrinsic immunogenicity with durable response to immune checkpoint blockade (22). EZH2 inhibition followed by STING agonism enhanced non-NE differentiation and T cell recognition of SCLC in a syngeneic mouse model. Treatment with EZH2 inhibitors can also improve chemosensitivity in SCLC via downregulation of SLFN11 (48, 49). Thus, utilizing EZH2 inhibition therapeutically in the context of tSCLC, to drive immunogenicity of non-NE tSCLC, or in combination with chemotherapy, are additional therapeutic possibilities worthy of further study.

The mechanistic basis for SCLC transformation in *EGFR*-mt LUAD is not fully understood. Loss of *RB1* and *TP53* is necessary but not sufficient to lead to SCLC transformation (5, 10). Loss of expression of EGFR is also observed through epigenetic silencing (50) (Figure 5, B–D). The lack of response to EGFR-TKI in tSCLC may be attributable to a decrease in EGFR expression upon transformation, similar to how transformed small cell carcinoma of the prostate reduces sensitivity to antiandrogen treatment because of loss of AR expression (19). However, our studies demonstrate that derepression of EGFR, either through epigenetic modulation or through ectopic expression alone, is not sufficient to restore EGFR dependence or sensitivity to EGFR inhibitors (Figure 5, E–H). How tSCLC evades EGFR pathway dependency upon SCLC transformation remains unknown and requires further study. Due to inactivation of *RB1* and *TP53*, *EGFR*-mt LUAD is stimulated to become NE and subsequently downregulate expression of EGFR (51). Even though the transformed cells become less addicted to EGFR signaling, our study suggests that they partially remain in an intermediate-transition cell state and that they retain sensitivity to downstream signaling of EGFR (Figure 7E). Moreover, the vast majority of transformed SCLCs after genotype-directed therapy have been described in *EGFR-*mt cancers, although such transformed SCLCs have also been reported for *ALK*- and *ROS1*-rearranged cancers (52–54). Whether this is a result of a greater number of patients treated with EGFR-targeted therapies or a higher propensity of *EGFR*-mt cancers to develop tSCLC remains to be determined, and whether similar biology described here can be extended to these other genomic contexts requires further study.

In summary, we present evidence that *EGFR*-mt tSCLC tumors have a plastic and heterogeneous nature consisting of both epithelial NE and mesenchymal non-NE cells. These 2 cell line populations not only are important for cell growth but also have differing drug sensitivities. Our findings highlight potentially unique features of *EGFR-*mt tSCLC compared with de novo SCLC, which will likely have future therapeutic implications.

## Methods

Additional methods details are in Supplemental Methods.

### Sex as a biological variable.

Across our studies, sex was not considered a biological variable affecting SCLC. To minimize aggressive interactions among cohoused mice, which could influence experimental results, we used mostly female animals.

### Cell cultures and reagents.

The following lung cancer cell lines were used: (SCLC) H69, H82, H209, Glc16, H1048, SW1271, H1882, H345, H526, H1417, H1105, DMS153 (LUAD) PC-9, HCC827, HCC4006. All cell lines except SW1271 were maintained in RPMI-1640 (Gibco) supplemented with 10% fetal bovine serum (FBS), 100 U/mL penicillin, and 100 μg/mL streptomycin (P/S) (Gibco). SW1271 was grown in DMEM (Gibco) with 10% FBS. Gefitinib, osimertinib, AZD8055, ZSTK474, MK2206, EPZ6438, GSK126, and compounds listed in Supplemental Table 6 were purchased from Selleck Chemicals. Stock solutions of all drugs were prepared at either 5 or 10 mmol/L in DMSO (Sigma) and stored at –80°C. Cell lines were authenticated by single tandem repeat analysis at the Molecular Biology Core Facilities in 2021 and tested negative for mycoplasma as determined the Mycoplasma Plus PCR Primer set (Agilent). LIF neutralizing antibody (AF-250-NA), IL-6 neutralizing antibody (AF-206-NA), and control antibody (AB-108-C) were purchased from R&D Systems. Human EGF recombinant protein was obtained from Life Technologies.

### Generation of a patient-derived cell line.

The malignant pleural effusions were obtained from a patient with *EGFR*-mt SCLC transformed tumors. The sample was subjected to red blood cell lysis using RBC Lysis Buffer (Boston BioProducts), and the cells were suspended either in RPMI-1640 with 10% FBS or in ACL4 media (Gibco) either with or without 10% FBS. We carefully monitored the appearance of the cells during serial passage to minimize the loss of intratumor heterogeneity. To establish NE and non-NE tSCLC cell lines, adherent cells were separated from floating aggregates, thoroughly washed with PBS, and passaged. To ensure the purity of non-NE population, the adherent cells were carefully washed with PBS prior to trypsinization to avoid culture of detached cells. For the NE population, floating aggregates were propagated without trypsinization in ultra-low-attachment plates. Following establishment, NE tSCLC cell lines were maintained in ultra-low-attachment plates, whereas non-NE tSCLC cell lines were cultured in regular tissue culture plates. DFCI112F and DFCI112Ad were maintained in RPMI-1640 with 10% FBS and P/S. DFCI190F and DFCI283F were grown in ACL4 media with P/S without serum. DFCI190Ad and DFCI283Ad were cultured in ACL4 media with 10% FBS and P/S. The number of passages the cells have undergone during the establishment of the cell lines is as follows: DFCI112F/Ad, passage 5; DFCI163F, passage 13; DFCI163Ad, passage 10; DFCI190F, passage 27; DFCI190Ad, passage 15; and DFCI283F/Ad, passage 20.

### Antibodies and immunoblotting.

Cells were grown and treated as described and lysed with RIPA buffer with Triton X-100 (Boston BioProducts) supplemented with protease and phosphatase inhibitors. Immunoblotting was performed according to the antibody manufacturers’ recommendations. For Rb detection, NE-PER Nuclear and Cytoplasmic Extraction kit (Thermo Fisher Scientific) was used for nuclear protein extraction. For histone H3 and H3K27me3 detection, proteins were isolated by acid extraction. The following antibodies were obtained from Cell Signaling Technology: anti-pEGFR (Tyr1068, #3777), EGFR (#2232), EGFR (E746-A750del specific, #2085), SYP (#4329), phospho-ERK1/2 (Thr202/Tyr204, #4370), ERK1/2 (#9102), phospho-Akt (Ser473, #4058), Akt (#9272), CD44 (#3578), NCAM (CD56, #3576), RB (#9309), Vimentin (#3932), ZEB1 (#3396), SNAIL (#3879), Lamin B1 (#12586), PARP (#9542), and H3 (#9733). Anti-ASCL1 (ab211327), NEUROD1 (ab60704), and H3K27me3 (ab6002) antibodies were purchased from Abcam. Anti-tubulin antibody (T9026) is from Sigma-Aldrich. Anti-INSM1 (sc-377428) and Hsp90 (sc-7947) antibodies are from Santa Cruz Biotechnology. Anti–β-actin antibody (A3854) is from Sigma-Aldrich.

### Immunohistochemistry.

The samples were fixed in 10% neutral-buffered formalin overnight at room temperature, transferred to 70% ethanol, embedded in paraffin, and sectioned at 5 μm for further staining. H&E staining was performed at the Department of Pathology at the Brigham and Women’s Hospital. The following antibodies were used for staining: INSM1 (Santa Cruz Biotechnology sc-271408), chromogranin A (Abcam ab15160), SYP (Thermo Fisher Scientific MA5-16402), Rb (BD Biosciences, 554136), vimentin (Cell Signaling Technology 5741), GFP (Abcam ab6556), and luciferase (Abcam ab181640).

### RNA-Seq (Figure 3, F and G; Supplemental Figure 7, A, B, and D–F; and Supplemental Figure 10B) cDNA library construction.

Total RNA was quantified using the Quant-iT RiboGreen RNA Assay Kit and normalized to 5 ng/μL. An aliquot of 200 ng for each sample was transferred into library preparation, which was an automated variant of the Illumina TruSeq Stranded mRNA Sample Preparation Kit. This method preserves strand orientation of the RNA transcript. It uses oligo dT beads to select mRNA from the total RNA sample. It is followed by heat fragmentation and cDNA synthesis from the RNA template. The resultant cDNA then goes through library preparation (end repair, base A addition, adapter ligation, and enrichment) using Broad-designed indexed adapters substituted in for multiplexing. After enrichment the libraries were quantified with qPCR using the KAPA Library Quantification Kit for Illumina Sequencing Platforms and then pooled equimolarly. The entire process is in 96-well format, and all pipetting is done by either Agilent Bravo or Hamilton Starlet.

### Illumina sequencing.

Pooled libraries were normalized to 2 nmol/L and denatured using 0.1N NaOH prior to sequencing. Flow cell cluster amplification and sequencing were performed according to the manufacturer’s protocols using either the HiSeq 2000 or HiSeq 2500. Each run was a 101 bp paired-end with an 8-base index barcode read. Data were analyzed using the Broad Picard Pipeline, which includes demultiplexing and data aggregation.

### RNA-Seq (Figure 5D).

Total RNA was isolated from cell lines or tissues using QIAGEN RNeasy kit. A total of 500 ng RNA was used to prepare libraries using the NEBNext Ultra RNA Library Prep Kit for Illumina. RNA quantity and quality were assessed on an Agilent 2100 Bioanalyzer. For all RNA-Seq, reads were sequenced on a NextSeq 500 instrument (Illumina).

### ScRNA-Seq.

The pleural effusion cells cryopreserved in 90% fetal bovine serum and 10% dimethyl sulfoxide were thawed and stained with human anti-CD45 (BioLegend 304008 clone ID HI30). Viable CD45-negative cells were sorted via FACS Melody (BD Biosciences). The cells were loaded onto a Chromium Controller (10x Genomics) per the manufacturer’s instructions. ScRNA libraries were generated using the Chromium Single Cell 3’ Reagent Kit with v3 Chemistry (10x Genomics). Quality control of sequencing libraries was determined using the High Sensitivity DNA Kit on a Bioanalyzer 2100 (Agilent). Finished libraries were sequenced using the HiSeq 2500 platform with paired-end 150 bp parameters (Novogene). Raw base calls were demultiplexed and converted to FASTQ files using the “bcl2fastq” command. Feature-barcode matrices were generated from FASTQ files using Cell Ranger v3.1.0 with default parameters and the GRCh38-3.0.0 reference transcriptome. Filtered feature-barcode matrices were used for downstream analysis with R toolkit Seurat (v.4.0.0) (55). Cells expressing fewer than 10 or more than 10,000 genes, and cells with more than 20% of UMIs (unique molecular identifiers) mapping to mitochondrial genes were removed from consideration. An additional minimum number of UMI threshold (750) was used, and the cells with fewer than 750 UMIs were removed. Additionally, genes that were expressed in fewer than 6 cells were filtered out. The filtered matrix was log-normalized with scaling factor 10,000, centered gene-wise, and subjected to dimensionality reduction using PCA on the highly varying genes. Clustering was performed on the top 10 principal components using the shared nearest neighbor algorithm as implemented in Seurat. The clusters were visualized using the UMAP algorithm. To measure enrichment of a given gene signature, AUCell R package (56) were applied.

### Plasmid construction and viral infection.

The expression plasmid or empty plasmid, a packaging plasmid (psPAX2, Addgene #12260), and an envelope plasmid (pMD2.G, Addgene #12259) were cotransfected into HEK293T cells. Viral supernatants were harvested at 48 hours and filtered through 0.45 μm filter; spinoculation was performed by spinning at 1,000*g* for 90 minutes. After 48 hours, cells were selected in puromycin (1 μg/mL). *EGFP* and WT or exon 19 del-*EGFR* were subcloned into pCW57.1 (Addgene #41393), a doxycycline-inducible lentiviral vector.

### Cell proliferation and growth assays and IncuCyte.

Inhibition of growth by targeted kinase inhibitors was evaluated using CellTiter-Glo luminescent assay according to the manufacturer’s instructions (Promega). Cells were plated either in 96-well plates at a density of 2,000–8,000 per well or in a 384-well plate at a density of 500–1,000 per well and treated either on the same day (for suspension cells) or on the following day (for adherent cells). At 120 hours after drug addition, cell viability was measured. For Figure 5, D and E, GFP-expressing DFCI112F and DFCI190F were plated into 96-well, clear, round-bottom, ultra-low-attachment plates (Corning 7007) and treated as indicated. The size of the spheroids in the wells was determined by the metric total green object integrated intensity (GCU × μm^2^/image) using the IncuCyte S3 live-cell analysis system (Essen Bioscience).

### Preparation of CM and ELISA.

CM were prepared by incubating the cells for 48 hours. Collected media were filtered through 0.22 μm filters. Human Cytokine/Chemokine Magnetic Bead Panel (MilliporeSigma HCYTMAG-60K-PX30) was used for multiplex ELISA. The Legend Max Human LIF ELISA kit (BioLegend 443507) was used for the detection of LIF.

### Implantation of cell lines into mice (Figure 4, C–E).

GFP-labeled NE cells (DFCI112F) and non-NE cells (DFCI112Ad, unlabeled in Figure 4C and luciferase-labeled in Figure 4, D and E) were injected in right flanks of nude mice (Figure 4C) or NSG mice (Figure 4, D and E) either as pure populations (NE 1 × 10^6^ cells, non-NE 2 × 10^5^ cells) or mixed at a ratio of 5:1 (1.2 × 10^6^ cells). Tumor sizes and body weights were measured twice weekly.

### Antitumor activity in vivo (Figure 7, C and D).

GFP-labeled NE cells (DFCI112F) and luciferase-labeled non-NE cells (DFCI112Ad) were mixed at a 4:1 (NE: non-NE) ratio (NE 1 × 10^6^ cells plus non-NE 2.5 × 10^5^ cells) and transplanted subcutaneously on the right flanks of 6- to 8-week-old female NRG mice. Tumors were allowed to establish to 219 ± 54 mm^3^ in size before randomization into vehicle-treated, trametinib-treated (1 mg/kg, PO qd), cisplatin-treated (4 mg/kg, IP qw), and combination-treated groups (1 mg/kg and 4 mg/kg, respectively) of 8 mice per group. Trametinib was formulated with 0.5% hydroxpropyl methyl cellulose and 0.2% Tween 80. Cisplatin (Fresenius Kabi) was obtained from the Dana-Farber Cancer Institute (DFCI) pharmacy and diluted with normal saline before use. Tumor volumes were determined from caliper measurements by using the formula V = (length × width^2^)/2. Tumor sizes and body weights were measured twice weekly.

### Statistics.

Statistical analysis was performed using GraphPad Prism. One-way or 2-way ANOVA was used to test significance for in vitro and in vivo studies. All in vitro data were shown as means ± SD, and in vivo data were shown as means ± SEM. *P* < 0.05 was considered statistically significant.

### Study approval.

All patients were treated at DFCI from 2011 to 2014 and provided written informed consent for the analysis of their clinical specimens, and the studies were approved by the Institutional Review Board at DFCI. The study methodologies conformed to the standards set by the Declaration of Helsinki. All in vivo studies were conducted at DFCI with the approval of the DFCI Institutional Animal Care and Use Committee in an Association for Assessment and Accreditation of Laboratory Animals–accredited vivarium.

### Data availability.

Data are available in public repositories, in the Supporting Data Values XLS file, or from the corresponding author upon request. The WES data (DFCI190F, 190Ad, 283F, 283Ad, and 163Ad) and the scRNA-Seq data (DFCI112, DFCI190, and DFCI283) were deposited in the NCBI GEO database under accession number GSE310962.

## Author contributions

Conceptualization was carried out by AO and PAJ. Methodology was developed by AO, XJM, KN, BFS, BKE, SW, IC, CX, HW, and PCG. Investigation was performed by AO, AV, XJM, NRM, KN, JJO, MX, BFS, BKE, CMM, SW, PC, SS, TT, CX, PHL, and LMS. Visualization was done by AO, AV, XJM, SW, PC, MAB, CX, and LMS. Project administration was managed by AO, HWL, PCG, LMS, MYT, and PAJ. Resources were provided by RMT, AEA, ESC, CLC, GRO, PCG, and CPP. Supervision was conducted by AO, MGO, DAB, MYT, and PAJ. Writing – original draft was prepared by AO, AV, and PAJ. Writing – review and editing was performed by all authors.

## Funding support

This work is the result of NIH funding, in whole or in part, and is subject to the NIH Public Access Policy. Through acceptance of this federal funding, the NIH has been given a right to make the work publicly available in PubMed Central.

- Dana-Farber/Harvard Cancer Center by an NCI Cancer Center Support Grant NIH 5 P30 CA06516.
- NCI R35CA220497 (PAJ).
- American Cancer Society (CRP-17-111-01-CDD) (PAJ).
- Gohl Family Lung Cancer Research Fund (PAJ).
- Goldstein Family Research Fund (PAJ).
- Magliocco Family Lung Cancer Research Fund (PAJ).
- Department of Defense (W81XWH-19-1-0613).
- NIH (R01CA240342) (HW).
- Expect Miracles Foundation.
- Robert A. and Renée E. Belfer Family Foundation.

## Supplementary Material

Supplemental data

Unedited blot and gel images

Supplemental table 1

Supplemental table 2

Supplemental table 3

Supplemental table 4

Supplemental table 5

Supplemental table 6

Supplemental table 7

Supporting data values

## Version 1 01/23/2026

Electronic publication
