# Supplementary material for: EGFR-mutant transformed small cell lung cancer harbors intratumoral heterogeneity targetable with MEK inhibitor combination therapy
Source: JCI Insight. 2026 Jan 23;11(2):e197008. doi: 10.1172/jci.insight.197008 (PMC12892894; doi:10.1172/jci.insight.197008)
Supplement: Supplemental data [file jciinsight-11-197008-s008.pdf]

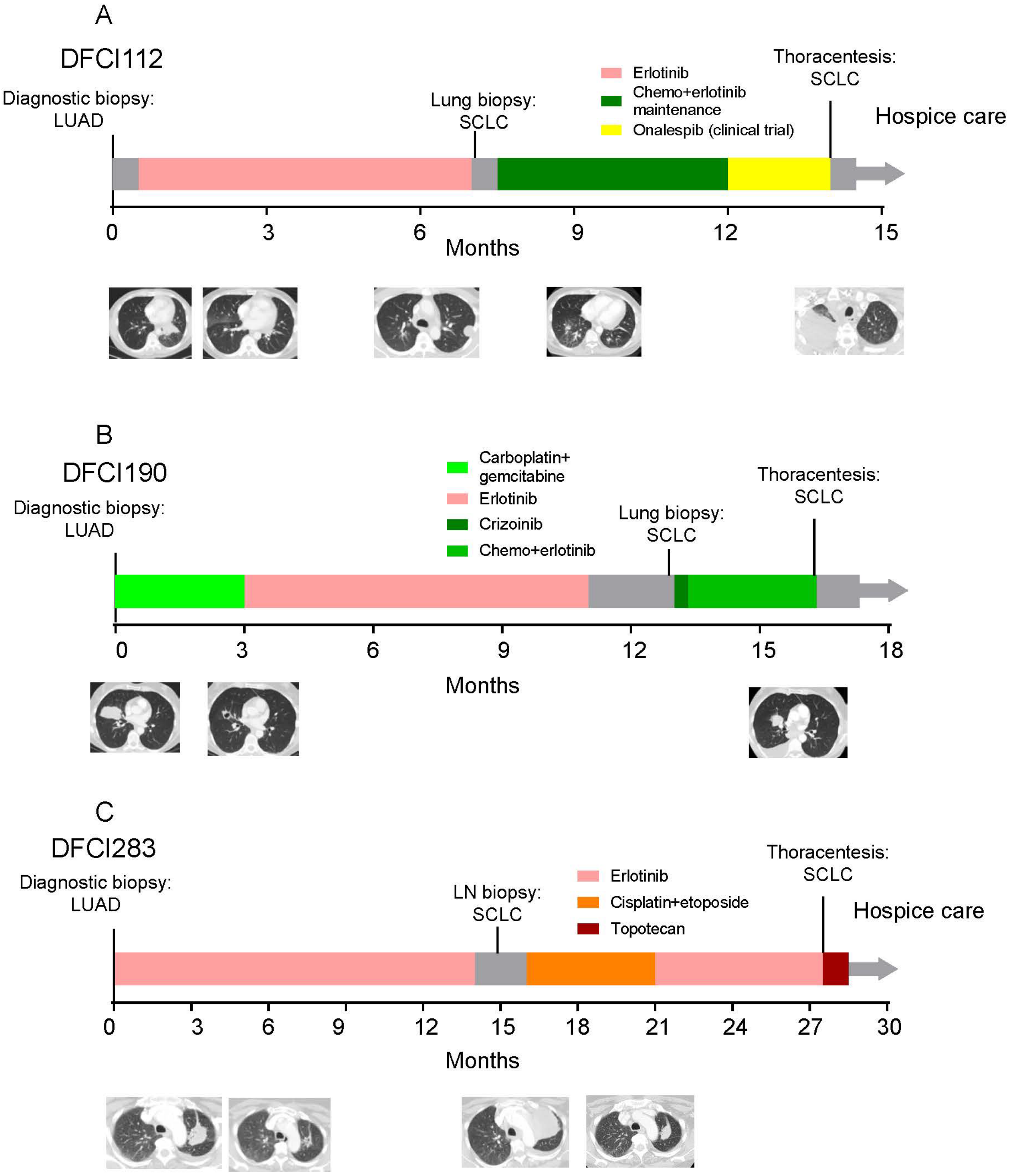
A
DFCI112
Diagnostic biopsy:
LUAD
Lung biopsy:
SCLC
Erlotinib
Chemo+erlotinib
maintenance
Onalespib (clinical trial)
Thoracentesis:
SCLC
Hospice care
0
3
6
9
12
15
Months
B
DFCI190
Diagnostic biopsy:
LUAD
Carboplatin+
gemcitabine
Erlotinib
Crizoinib
Chemo+erlotinib
Lung biopsy:
SCLC
Thoracentesis:
SCLC
0
3
6
9
12
15
18
Months
C
DFCI283
Diagnostic biopsy:
LUAD
LN biopsy:
SCLC
Erlotinib
Cisplatin+etoposide
Topotecan
Thoracentesis:
SCLC
Hospice care
0
3
6
9
12
15
18
21
24
27
30
Months

D

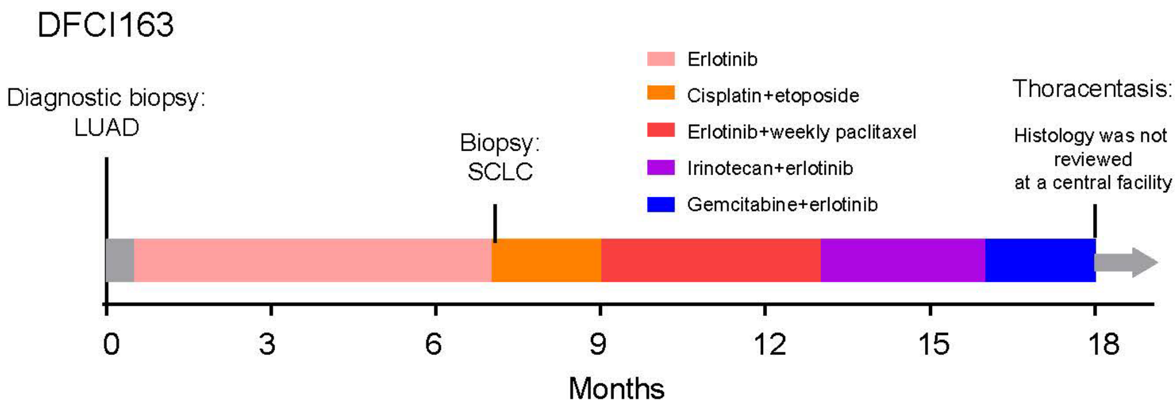

Figure S1. The clinical course of patients with a history of SCLC transformation. (**A**) DFCI112 (**B**) DFCI190 (**C**) DFCI283 (**D**) DFCI163.

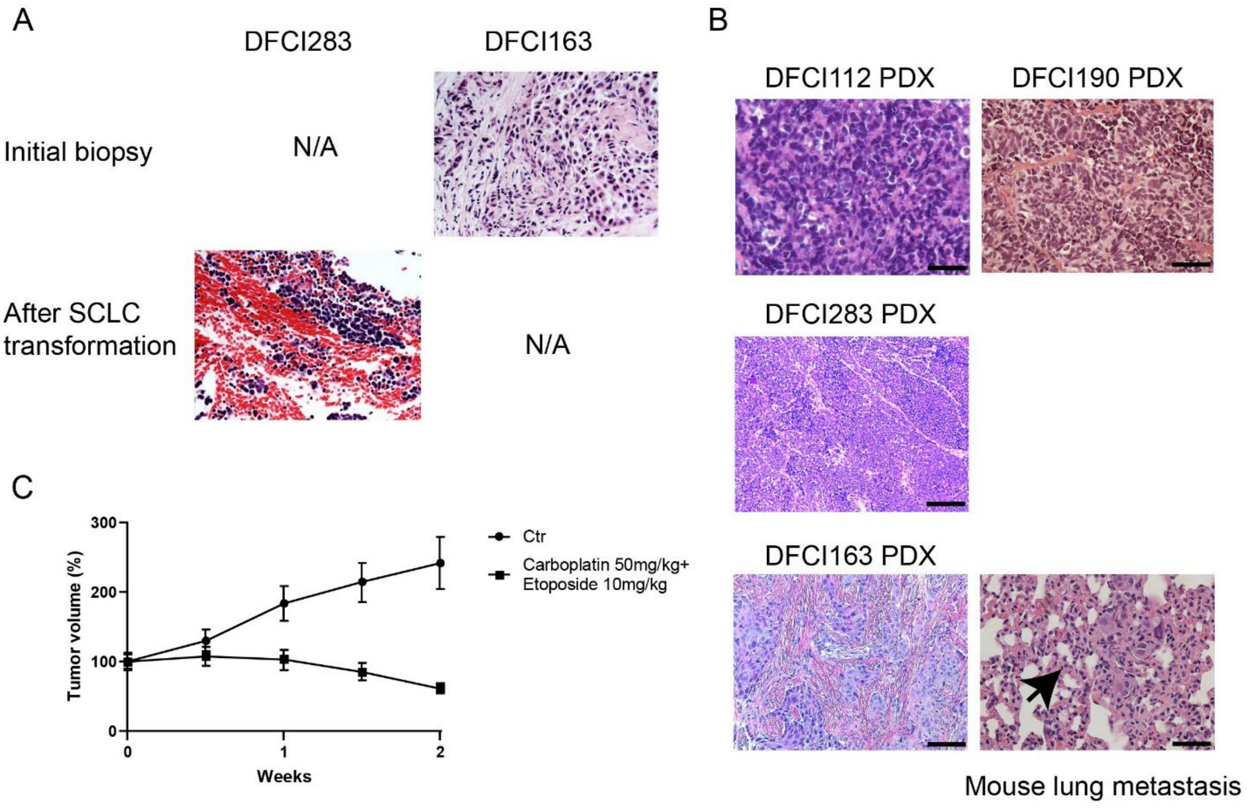

Figure S2. Characterization of cell line and xenograft models established from SCLC transformed patients. (**A**) H&E staining (200X) of either pre- or post-EGFR-TKI treatment lung biopsies from DFCI163 and DFCI283. N/A: non-available (although histology was reviewed by pathologists, histopathological images were not available). (**B**) H&E staining of patient-derived xenografts (PDX) of DFCI112, DFCI190, DFCI283 and DFCI163. 400X, scale bar, 50 μm. (**C**) Tumor size measurement of DFCI112 xenograft tumors treated with vehicle or combination of carboplatin (50 mg/kg, i.p. twice a week) and etoposide (10 mg/kg, i.p. once per day for five consecutive days) with indicated treatment period. 15 mice per condition.

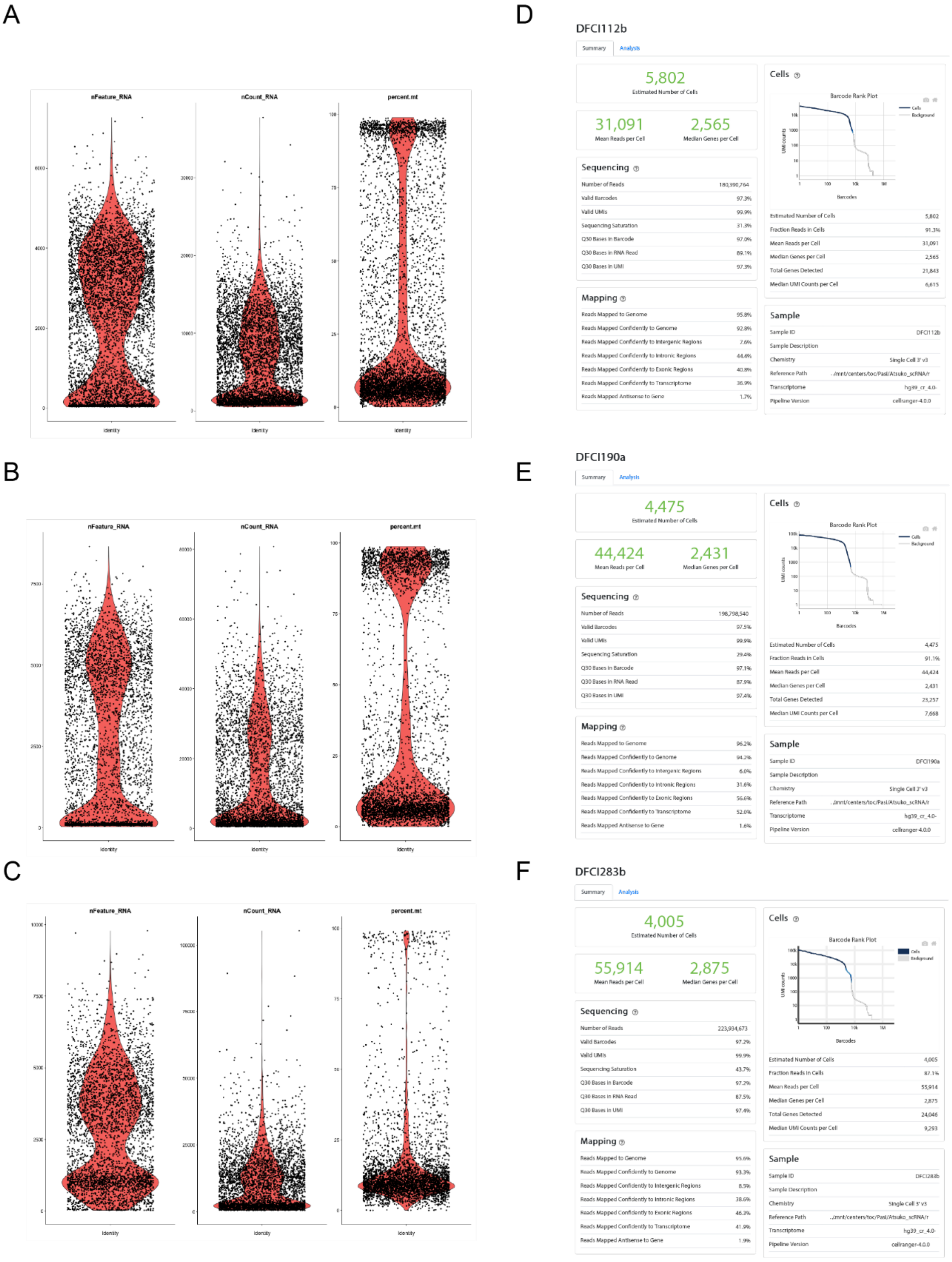

Supplementary Figure 3

G

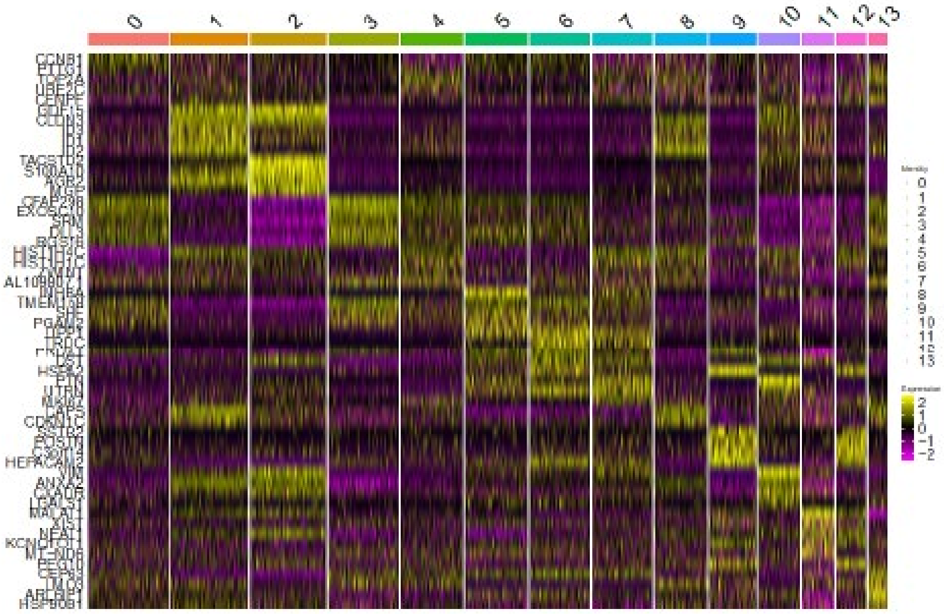

H

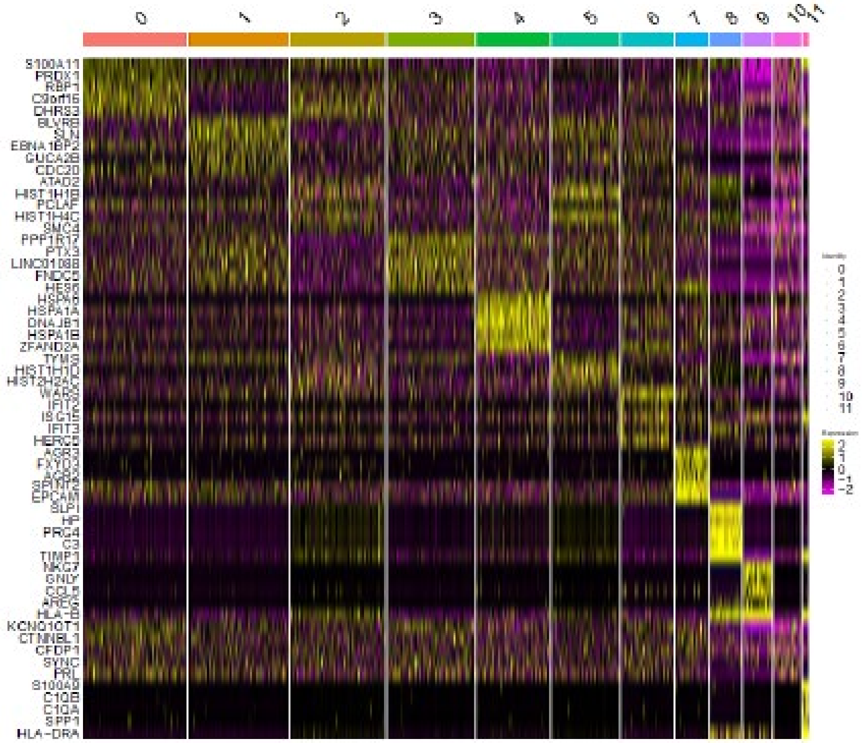

Supplementary Figure 3

I

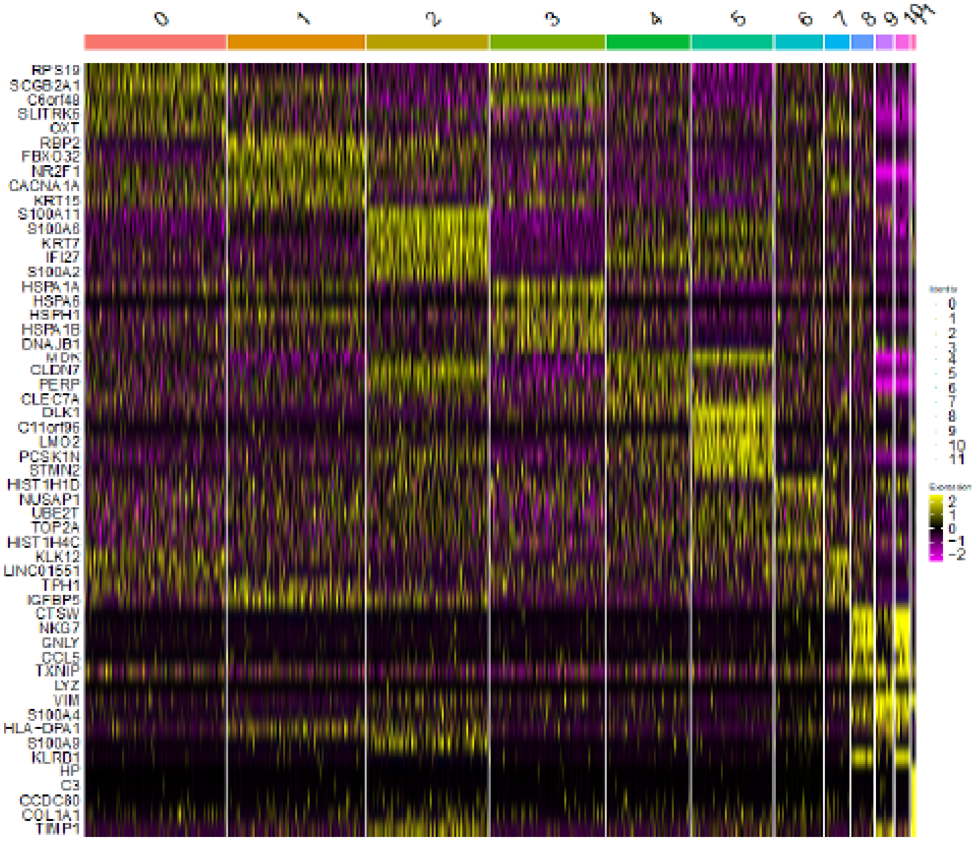

J

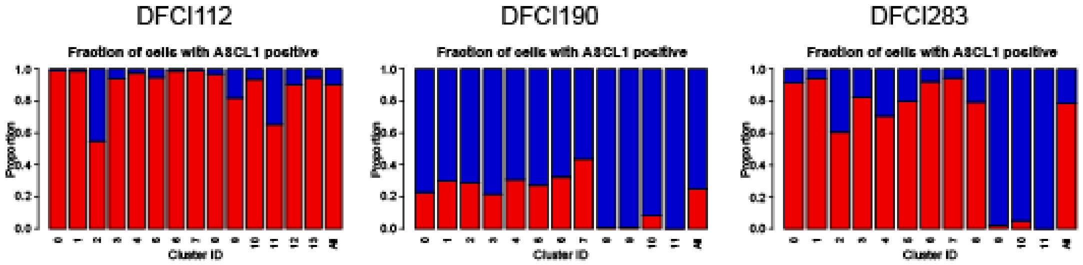

Supplementary Figure 3

K

DFCI112

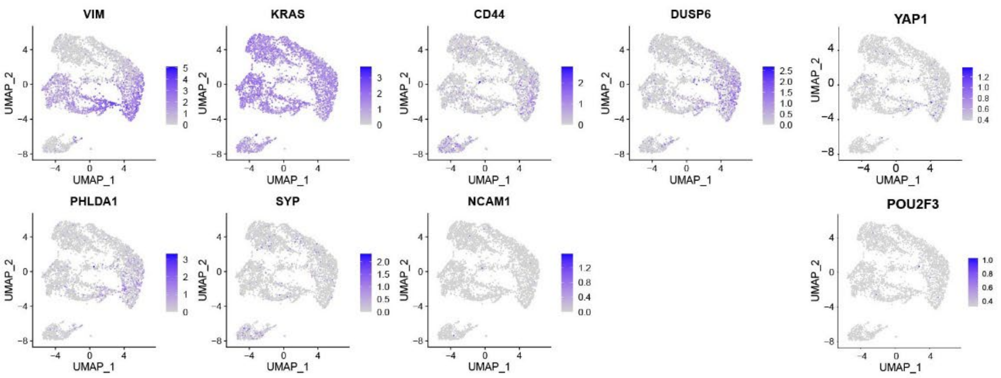

L

DFCI190

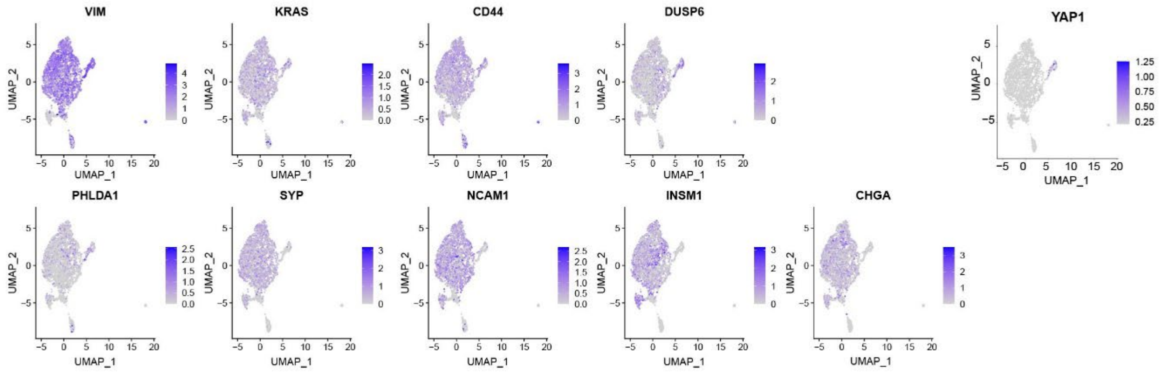

M

DFCI283

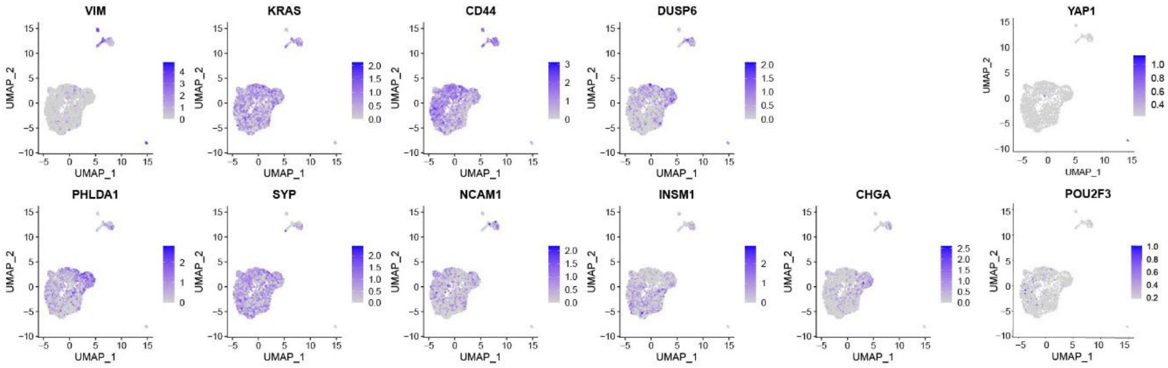

Supplementary Figure 3

N

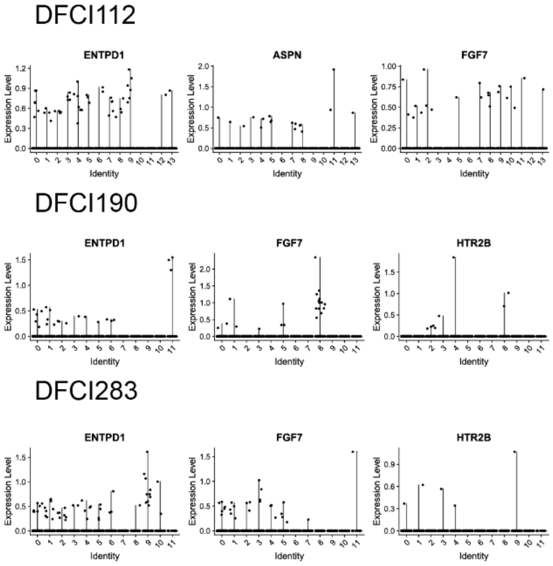

O

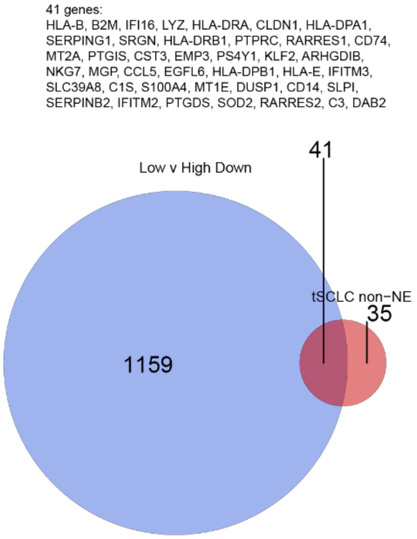

P

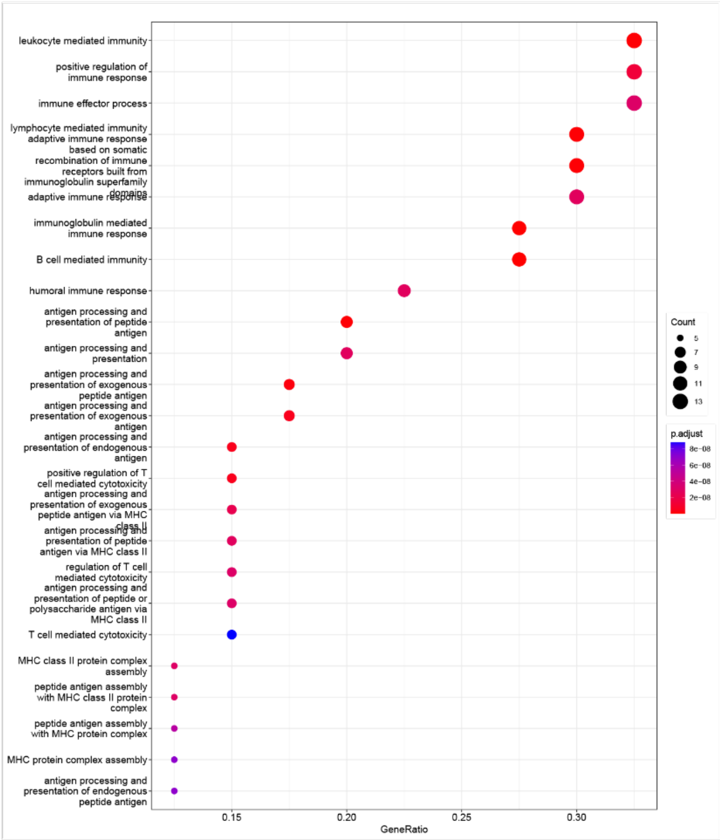

Supplementary Figure 3

Figure S3. Single cell-analyses reveal intratumor heterogeneity in pleural effusions of patients with SCLC transformation. (**A-F**) Quality control (QC) metrics of scRNA-seq datasets of DFCI112 (**A, D**), DFCI190 (**B, E**), and DFCI283 (**C, F**). (**G-I**) Heatmaps of the top five differentially expressed genes in each cluster of DFCI112 (G), DFCI190 (H), and DFCI283 (**I**). (**J**) Bar plots of the proportion of *ASCL1* positive fractions of the cells in DFCI112, DFCI190, and DFCI283. (**K-M**) UMAP feature plots of marker genes. (**K**) DFCI112, (**L**) DFCI190, (**M**) DFCI283. (**N**) The violin plots for representative lineage marker genes (*ENTPD1, ASPN, FGF7, HTR2B*). (**O**) The Venn diagram of shared and differentially expressed genes in RNA-seq between traditional non-NE SCLC and non-NE tSCLC. (**P**) GSEA of differentially expressed gene sets comparing non-NE SCLC and non-NE tSCLC.

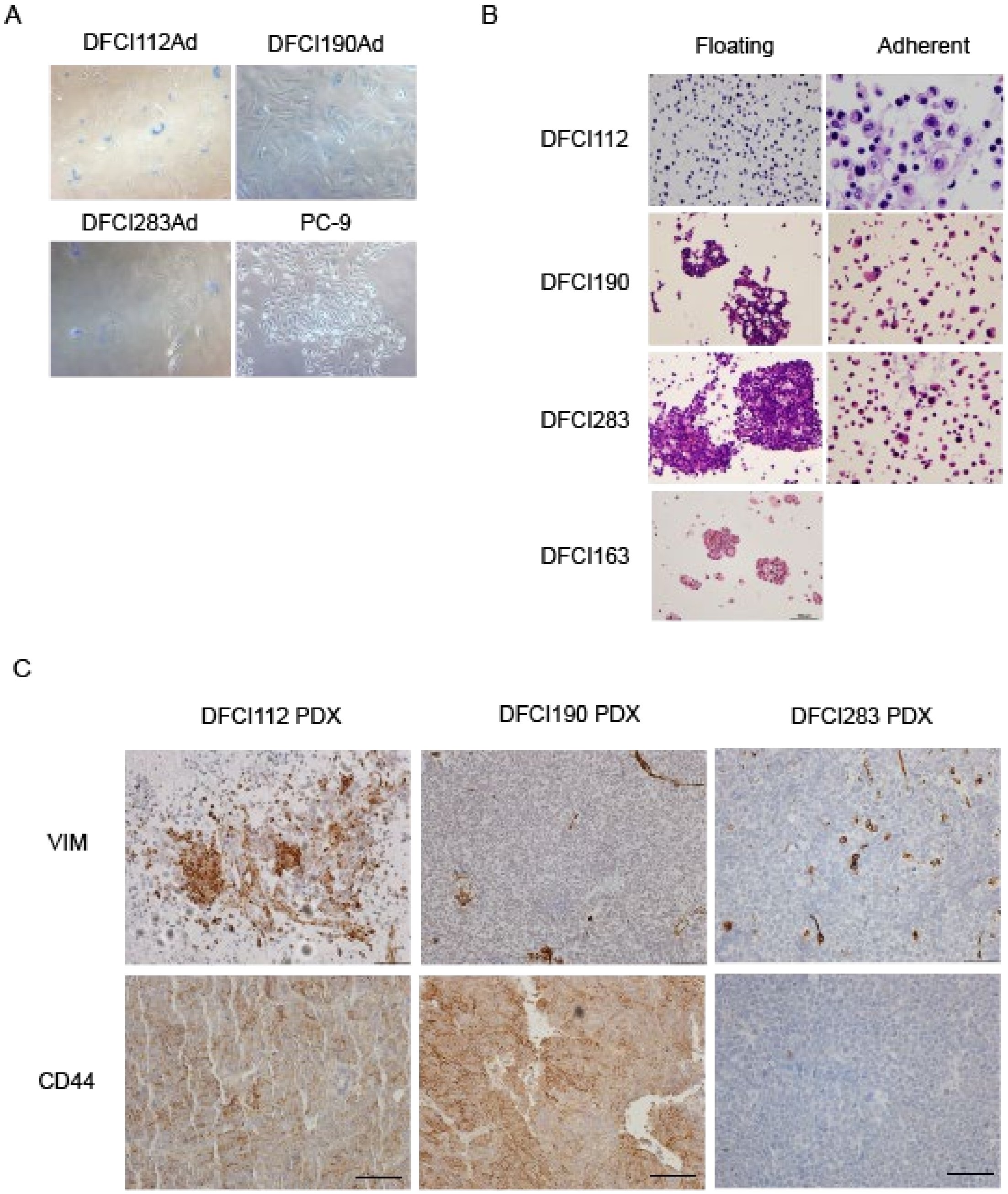

Supplementary Figure 4

Figure S4. In vitro and in vivo tSCLC models recapitulate the heterogeneity of primary cancer cells from tSCLC patients. (**A**) Senescence-associated β-galactosidase staining of non-NE tSCLC cells and PC-9. (**B**) H&E staining of fixed cell pellets of tSCLC and tLUAD cell lines. (**C**) IHC images for vimentin (VIM) and CD44 of patient-derived xenografts (PDX) of DFCI112, DFCI190, and DFCI283. DFCI112 and DFCI190; 200X, scale bar, 100 μm. DFCI283; 400X, scale bar, 50 μm

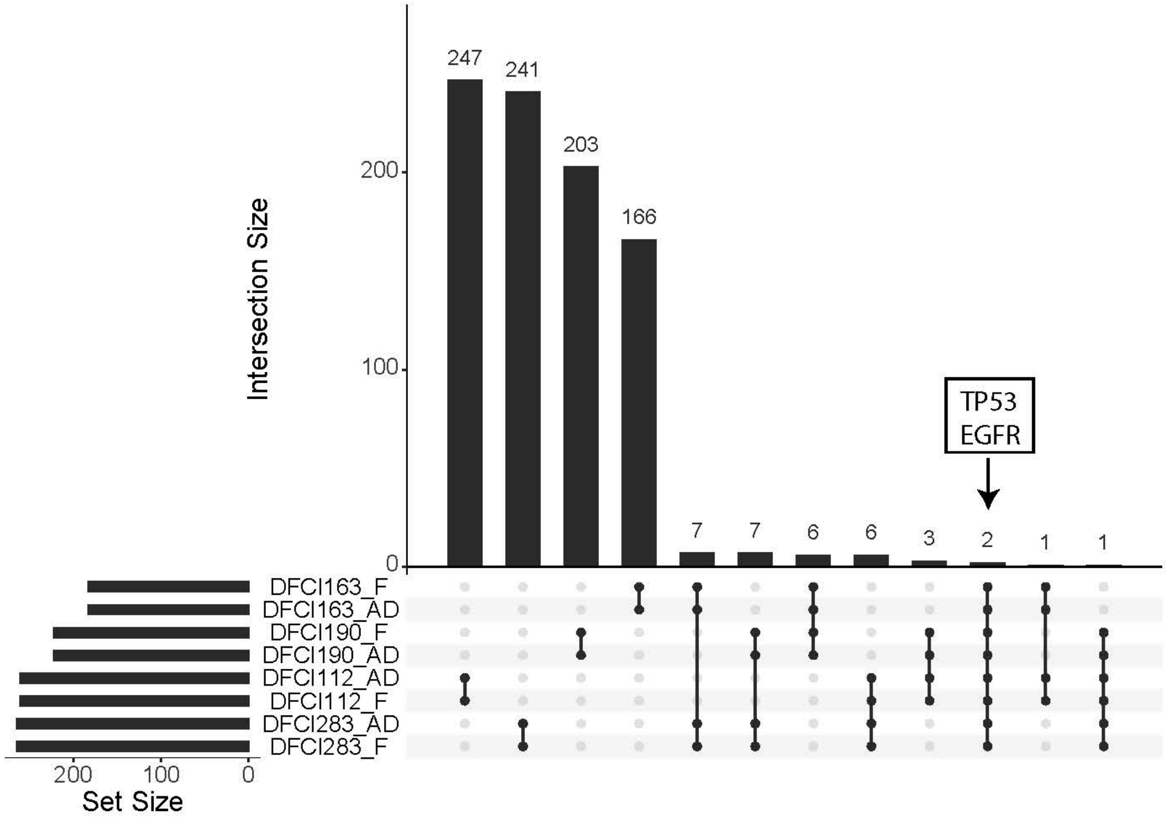

Figure S5. Numbers of shared mutations across tSCLC and tLUAD cell lines identified by whole-exome sequencing.

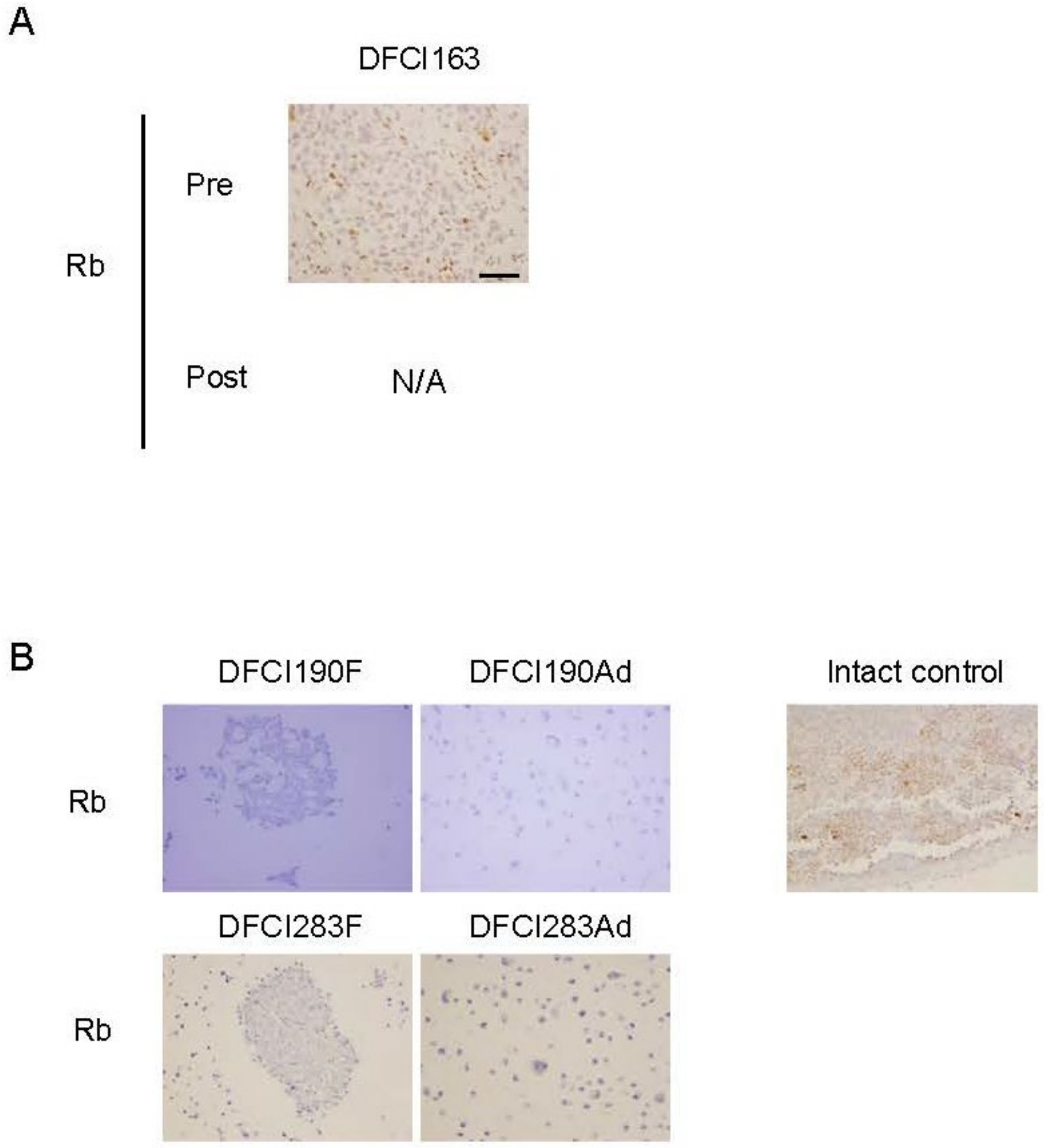

Figure S6. Rb expression is absent before and after EGFR-TKI treatment in the patients with SCLC transformation. (**A**) Rb IHC staining (400X) of pre-EGFR-TKI treatment lung biopsies from DFCI163. The staining of post-treatment was not available (N/A). Scale bar, 50 µm. (**B**) Rb IHC staining (200X-400X) of cell pellets of DFCI190F/Ad and DFCI283F/Ad.

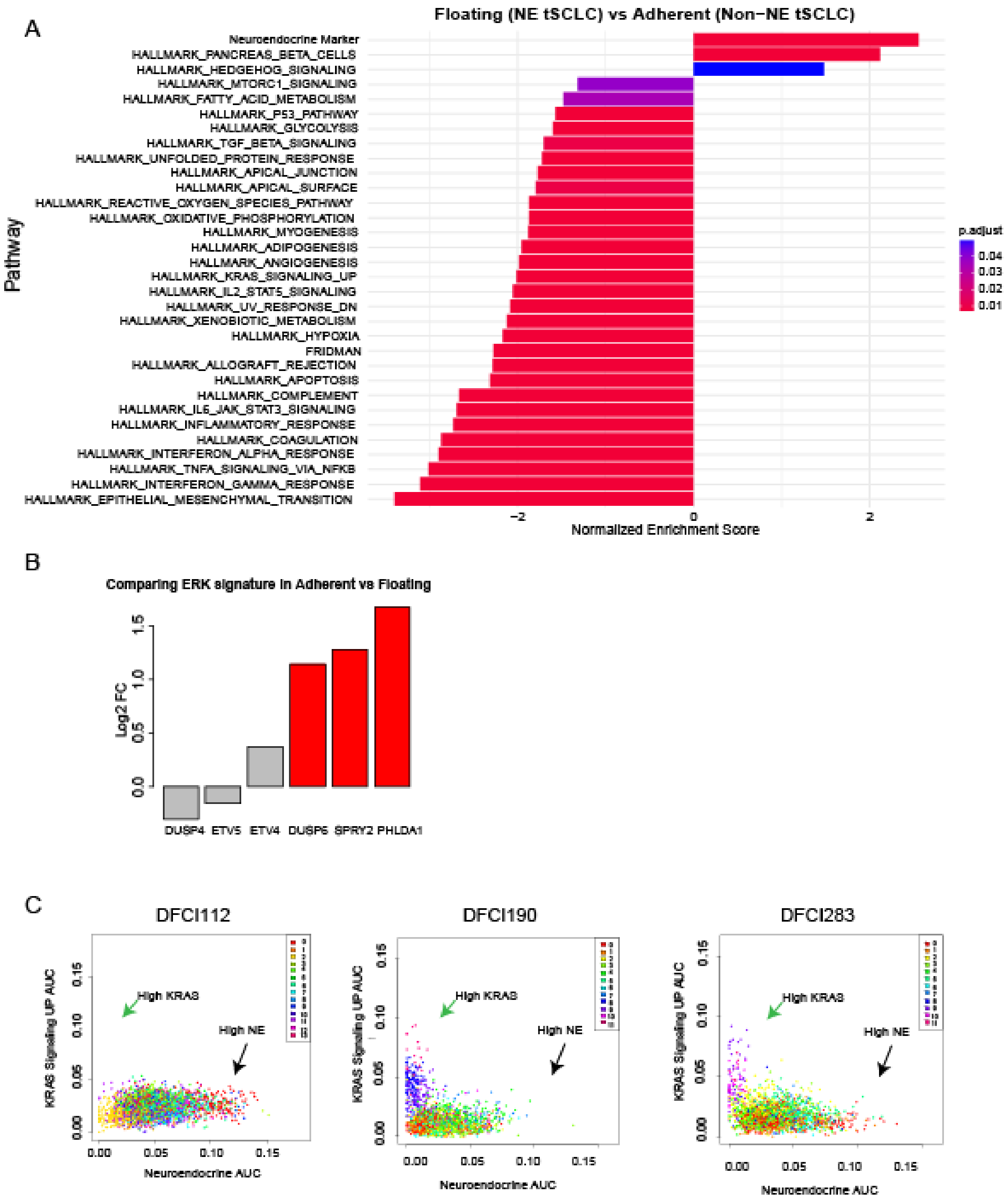

Supplementary Figure 7

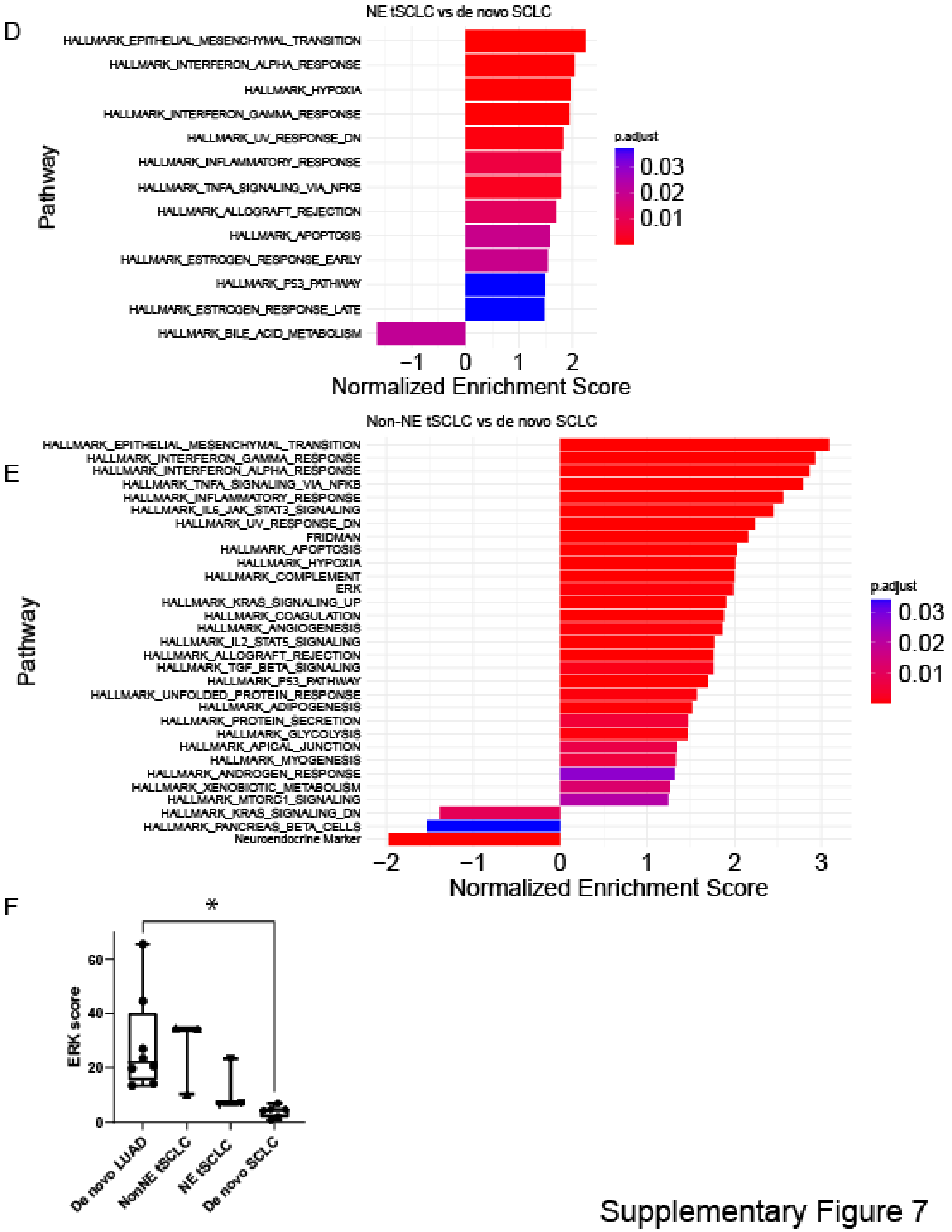

Supplementary Figure 7

Figure S7. Non-NE tSCLC is enriched with an EMT and the KRAS activation gene signature. (**A**) Bar plot of normalized enriched scores (NES) of pathways for comparing floating with adherent cell lines. Pathway analysis was performed using GSEA approach based on the gene expression fold-changes between NE and non-NE groups. (**B**) The log2 FC values for ERK signature. (**C**) The scatter plots of enrichment score for NE vs KRAS signaling signature for DFCI112, DFCI190, and DFCI283. (**D, E**) Bar plots of NES of pathways for comparing NE tSCLC with *de novo* SCLC (**D**) and non-NE tSCLC with *de novo* SCLC (**E**). (**F**) Graph showing the ERK activity score (*PHLDA1, SPRY2, SPRY4, ETV4, ETV5, DUSP4, DUSP6, CCND1, EPHA2, and EPHA4*) comparing *de novo* LUAD (n = 8), non-NE tSCLC (n = 3), NE tSCLC (n = 3), and *de novo* SCLC (n = 6) cell lines. The cell lines used for this analysis are listed in Supplementary Table S6. *= p ≤0.05, by one-way ANOVA with Turkey's multiple comparisons test.

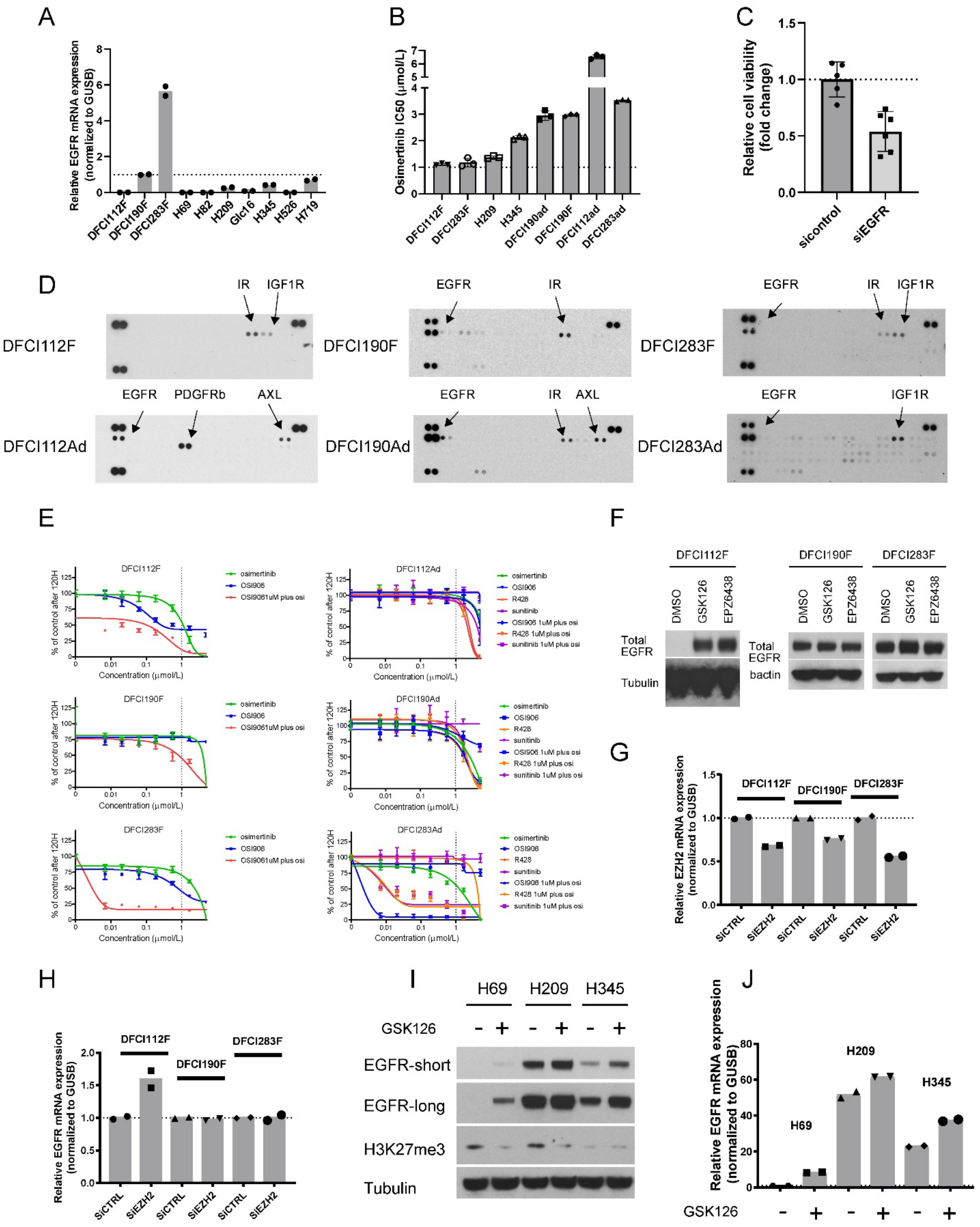

Supplementary Figure 8

Figure S8. Wide spectrum of EGFR expression and dependency in transformed and *de novo* SCLC. (**A**) Quantitative PCR analysis of *EGFR* mRNA expression levels in various SCLC cell lines as indicated after normalization to *GUSB* and relative to that in DFCI190F (n=2, technical replicate, mean). (**B**) A comparison of IC_{50} values of osimertinib for tSCLC and SCLC cell lines after 120 hours of treatment (n=3, technical replicate, mean±SD). (**C**) Relative cell viability of DFCI283F treated either with control siRNA or *EGFR* specific siRNA for 72 hours. The cell viability was assessed by CTG (n=6, technical replicates, mean±SD). (**D**) Phospho-RTK array of NE tSCLC and non-NE tSCLC cell lysates. (**E**) Drug response curve of NE tSCLC cells (left) and non-NE tSCLC cells (right) treated with various concentrations of inhibitors for five days. (**F**) Western blot analysis of DFCI112F, as compared with DFCI190F and DFCI283F pretreated either with DMSO, 10 µmol/L of GSK126 or EPZ6438 for 10 days with respective loading controls. Additional lysate (50 µg) from an independent experiment was used to validate the difference in EGFR levels in DFCI112F, using tubulin as a loading control. (**G**, **H**) qPCR analysis of *EZH2* (**G**) and *EGFR* mRNA (**H**) expression levels in DFCI112F, DFCI190F and DFCI283F treated with control siRNA or *EZH2* specific siRNA for 72 hours. Ratios were calculated after normalization to *GUSB* and relative to the mRNA expression level in control siRNA treated cells (n=2, technical replicate, mean). (**I**) Western blot analysis of H69, H209 and H345 pretreated either with DMSO or 5 µmol/L of GSK126 for 11 days. The blots were probed with antibodies against total EGFR, H3K27me3 and tubulin (loading control). (**J**) qPCR analysis of *EGFR* mRNA expression levels in H69, H209 and H345 pretreated with 5 µmol/L of GSK126 for seven days. Ratios were calculated after normalization to *GUSB* and relative to the mRNA expression level in DMSO treated cells (n=2, technical replicate, mean).

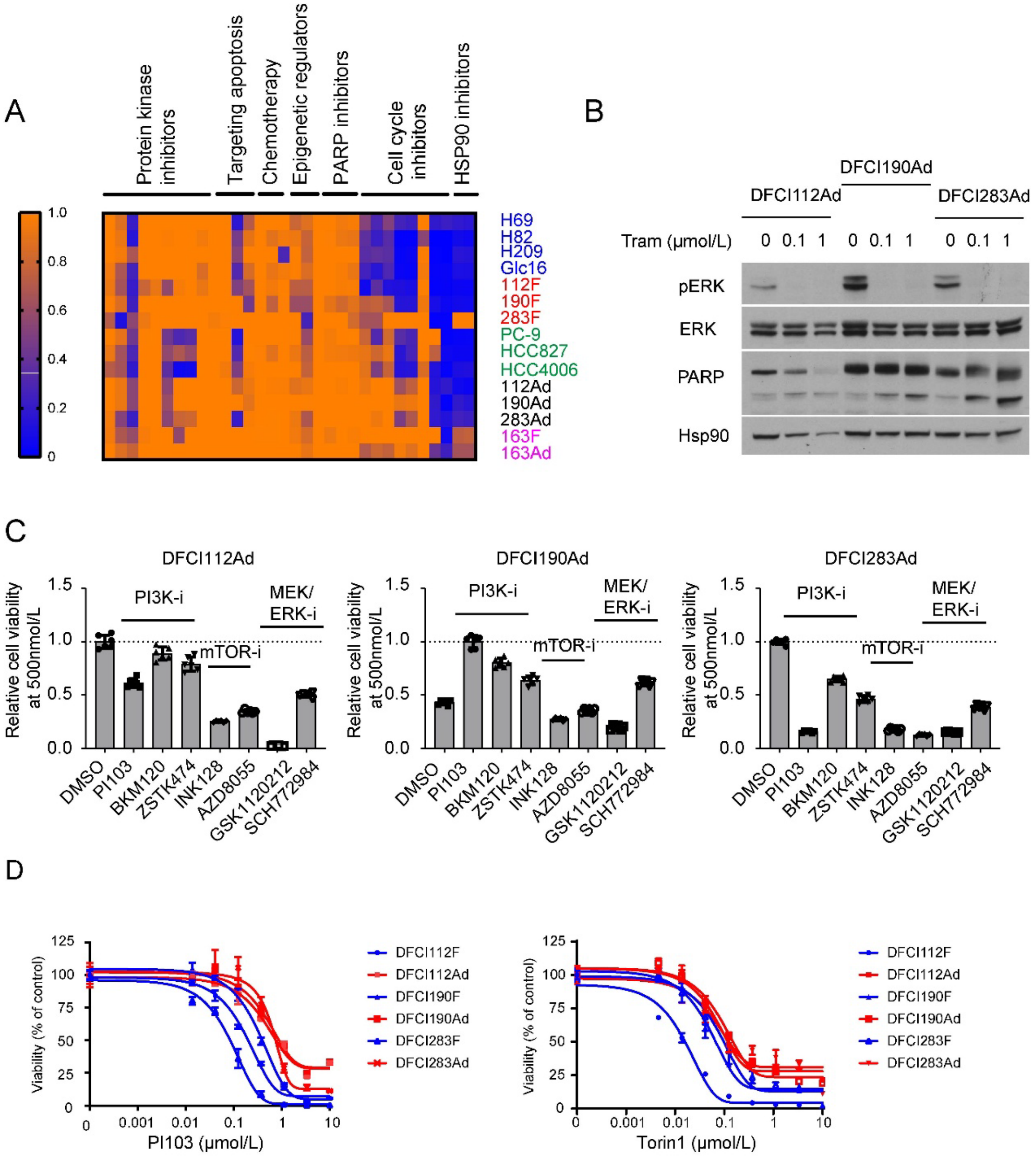

Supplementary Figure 9

Figure S9. The difference in pathway dependency between NE and non-NE tSCLC cell lines. (**A**) Heatmap of cell viability after five days treatment with 100 nmol/L of each single agent (columns) per various lung cancer cell lines (raws) relative to DMSO control. The commercially available agents used in this analysis are listed in Supplementary Table S7. (**B**) Western blot analysis of DFCI112Ad, DFCI190Ad and DFCI283Ad treated either with the indicated concentration of DMSO or trametinib for 48 hours. The blots were probed with antibodies against pERK, ERK, PARP or Hsp90 (loading control). (**C**) The comparison of cell viability in non-NE tSCLC cell lines relative to DMSO control in the presence of various PI3K/mTOR an MEK/ERK inhibitors at the concentration of 500 nmol/L. (**D**) Dose response curve of PI103 and torin1 in tSCLC cell lines. The cell viability was assessed by CTG after 120 hours (n=6, technical replicate, mean±SD)

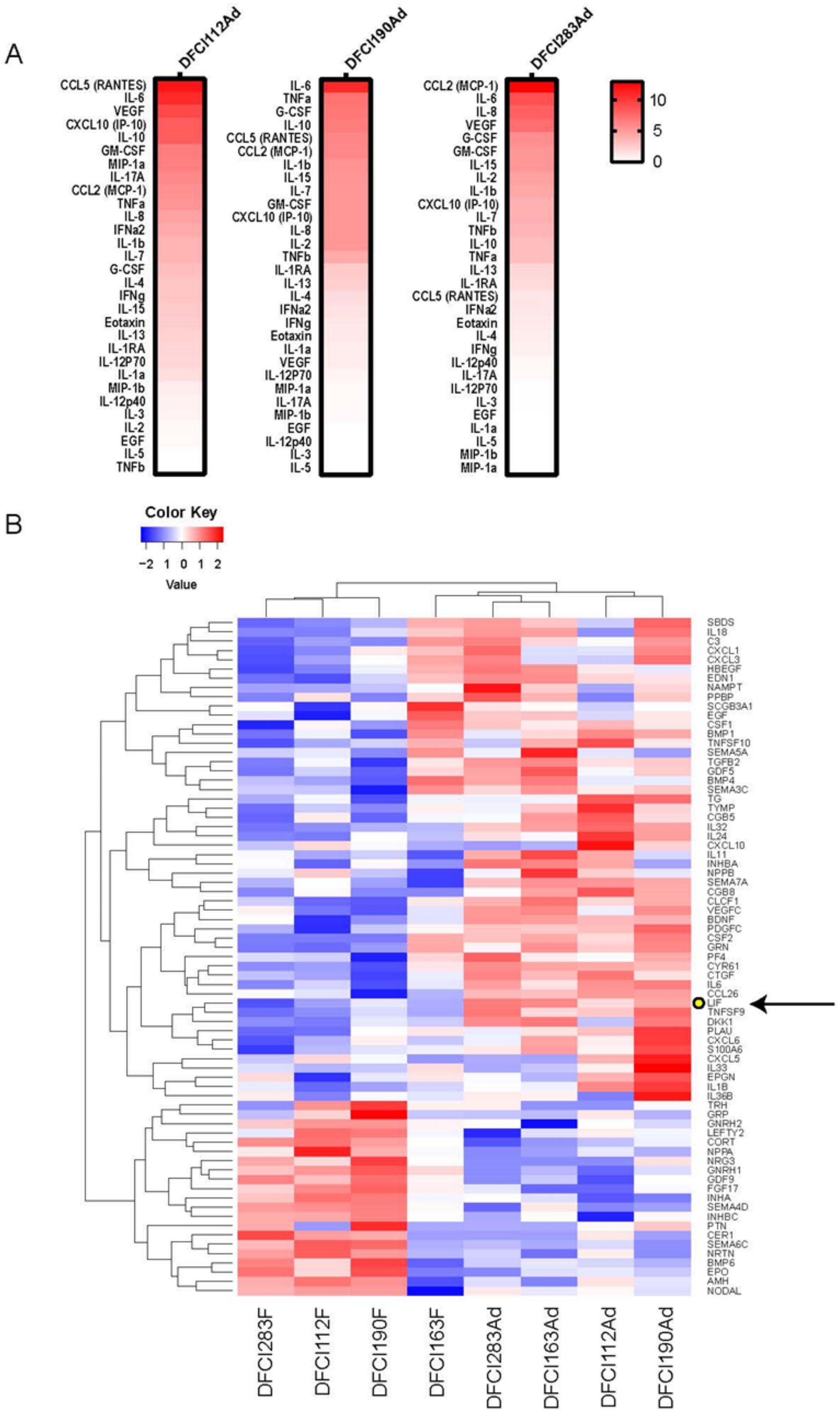
A
DFCI112Ad
CCL5 (RANTES)
IL-6
VEGF
CXCL10 (IP-10)
IL-10
GM-CSF
MIP-1a
IL-17A
CCL2 (MCP-1)
TNFa
IL-8
IFNa2
IL-1b
IL-7
G-CSF
IL-4
IFNg
IL-15
Eotaxin
IL-13
IL-1RA
IL-12P70
IL-1a
MIP-1b
IL-12p40
IL-3
IL-2
EGF
IL-5
TNFb
DFCI190Ad
IL-6
TNFa
G-CSF
IL-10
CCL5 (RANTES)
CCL2 (MCP-1)
IL-1b
IL-15
IL-7
GM-CSF
CXCL10 (IP-10)
IL-8
IL-2
TNFb
IL-1RA
IL-13
IL-4
IFNa2
IFNg
Eotaxin
IL-1a
VEGF
IL-12P70
MIP-1a
IL-17A
MIP-1b
EGF
IL-12p40
IL-3
IL-5
DFCI283Ad
CCL2 (MCP-1)
IL-6
IL-8
VEGF
G-CSF
GM-CSF
IL-15
IL-2
IL-1b
CXCL10 (IP-10)
IL-7
TNFb
IL-10
TNFa
IL-13
IL-1RA
CCL5 (RANTES)
IFNa2
Eotaxin
IL-4
IFNg
IL-12p40
IL-17A
IL-12P70
IL-3
EGF
IL-1a
IL-5
MIP-1b
MIP-1a
10
5
0
B
Color Key
−2 1 0 1 2
Value
SBDS
IL18
C3
CXCL1
CXCL3
HBEGF
EDN1
NAMPT
PPBP
SCGB3A1
EGF
CSF1
BMP1
TNFSF10
SEMA5A
TGFB2
GDF5
BMP4
SEMA3C
TG
TYMP
CGB5
IL32
IL24
CXCL10
IL11
INHBA
NPPB
SEMA7A
CGB8
CLCF1
VEGFC
BDNF
PDGFC
CSF2
GRN
PF4
CYR61
CTGF
IL6
CCL26
LIF
TNFSF9
DKK1
PLAU
CXCL6
S100A6
CXCL5
IL33
EPGN
IL1B
IL36B
TRH
GRP
GNRH2
LEFTY2
CORT
NPPA
NRG3
GNRH1
GDF9
FGF17
INHA
SEMA4D
INHBC
PTN
CER1
SEMA6C
NRTN
BMP6
EPO
AMH
NODAL
DFCI283F
DFCI112F
DFCI190F
DFCI163F
DFCI283Ad
DFCI163Ad
DFCI112Ad
DFCI190Ad

Figure S10. Cytokine and growth factor expression profiles in non-NE tSCLC. (**A**) Cytokine expression profiles of DFCI112Ad, DFCI190Ad and DFCI283Ad. The expression level of 30 analytes was evaluated using Milliplex MAP human cytokine/chemokine panel kit. Data represent mean ± SD (n=3, technical replicates). (**B**) RNA-seq heatmap of growth factor genes in tSCLC and tLUAD cell lines. LIF is indicated by the arrow.

Table S1. The details of the patient-derived tSCLC and tLUAD cell lines used in this study

Table S2. The details of shared mutations across tSCLC and tLUAD cell lines (WES)

Table S3. Whole exome sequencing results of tSCLC and tLUAD cell lines

Table S4. The details of mutations in *RB1* in tSCLC and tLUAD

Table S5. Bulk RNA sequencing results of tSCLC and tLUAD cell lines

Table S6. A list of the cell lines used in PCA (Fig. 3F) and ERK activity score analysis (Fig. S7F)

Table S7. A list of commercially available compounds used for drug screening (Fig. 6A, Fig. S9A)

Supplemental Methods

Patient derived xenograft (PDX) establishment (Fig. S2B, S2C, and S4C)

To establish the tSCLC PDX models, approximately 5 x 10^6 cells derived from pleural effusion (DFCI112, DFCI283, and DFCI163) or tumor biopsies (DFCI190) were implanted subcutaneously in 8-week-old female NSG mice and tumor growth was monitored by caliper measurements. Once tumors grew to a size of 1 cm^3, tumors were isolated and cut into pieces of approximately 2 x 2 x 2 mm and transplanted subcutaneously in additional NSG mice. Tumors were passaged for no more than five times. Samples from all passages were viably frozen in liquid nitrogen and used for further experiments. The tumor fidelity from various passages was confirmed by H&E staining.

RNA isolation and quantitative real time-PCR (qPCR)

RNA was extracted using RNeasy kit (Qiagen). cDNA was generated from 1 μg of RNA using QuantiTect Reverse Transcription Kit (Qiagen). qPCR was performed and analyzed on Step One Plus real-time PCR System using TaqMan probes (Applied Biosystems) for RB1 (Hs01078066), LIF (Hs01055668), EGFR (Hs01076078) and EZH2 (Hs00544830). GUSB was used as a housekeeping gene.

Senescence-associated β−galactosidase staining

DFCI112Ad, DFCI190Ad, DFCI283Ad were plated into 12-well plates at 30,000 cells/well. PC-9 was plated at 60,000 cells/well. The cells were stained using senescence β−galactosidase staining kit (Cell Signaling Technology #9860) according to manufacturer's protocol.

OncoPanel

tSCLC cell lines were analyzed comprehensively by targeted next generation sequencing (OncoPanel (1)). The OncoPanel assay surveys exonic DNA sequences of 275 cancer genes and 91 introns across 30 genes for rearrangement detection. DNA was isolated from tSCLC cell lines using DNeasy Blood & Tissue Kit (Qiagen) and analyzed by massively parallel sequencing using a solution-phase Agilent SureSelect hybrid capture kit and an Illumina HiSeq 2500 sequencer.

Whole exome sequencing

Agilent Exomes

Starting with 250 ng input DNA, samples are quantified using a PicoGreen assay and diluted to a working stock volume and concentration (2 ng/µL in 50 µL), then libraries are constructed and sequenced on Illumina HiSeq 2500 with the use of 76-bp paired-end reads. Output from Illumina software is processed by the Picard data-processing pipeline to yield BAM files containing well-calibrated, aligned reads. All process steps are performed using automated liquid handling instruments, and all sample information tracking is performed by automated LIMS messaging.

Library Construction

Libraries are then constructed using the protocol described in Fisher et al. with several modifications: first, initial genomic DNA input into shearing has been reduced from 3 µg to 100 ng in 50 µL of solution. Second, for adapter ligation, Illumina paired end adapters have been replaced with palindromic forked adapters with unique 8 base index sequences embedded within the adapter. These index sequences enable pooling of libraries prior to sequencing. Third, custom sample preparation kits from Kapa Biosciences are now used for all enzymatic steps of the library construction process.

In-solution hybrid selection

In-solution hybrid selection was performed as described by Fisher et al (2).

Preparation of libraries for cluster amplification and sequencing

Following sample preparation, libraries are quantified using PicoGreen. Based on PicoGreen quantification, libraries are normalized to equal concentration and pooled by equal volume. Library pools are then quantified using a Sybr Green-based qPCR assay, with PCR primers complementary to the ends of the adapters (kit purchased from Kapa Biosciences). After qPCR quantification, library pools are normalized to 2 nmol/L, denatured using 0.2 N NaOH, and diluted to 20 pmol/L, the working concentration for downstream cluster amplification and sequencing. Denatured library pools are spread across the number of sequencing lanes required to hit target coverage for all samples.

Cluster amplification and sequencing

Cluster amplification and sequencing of denatured templates are performed according to the manufacturer's protocol (Illumina) using HiSeq 2500 cluster amplification kits, flowcells, Sequencing-by-Synthesis kits, Multiplexing Sequencing Primer kits, and the latest version of Illumina's RTA software. 76 bp paired end reads, with additional cycles added to read molecular index sequences, are performed. Output from Illumina software is processed by the Picard data-processing pipeline to yield BAM files containing well-calibrated, aligned reads.

ATAC-sequencing

Fifty thousand cells were resuspended in 1ml of cold ATAC-seq resuspension buffer (RSB) (10 mmol/L Tris-HCl (pH 7.4), 10 mmol/L NaCl and 3 mmol/L $MgCl_2$ in water). Cells were centrifuged at maximum speed for 10min in a prechilled (4°C) fixed-angle centrifuge. After centrifugation, supernatant was carefully aspirated. Cell pellets were then resuspended in 50 μl of

ATAC-seq RSB containing 0.1% NP40, 0.1% Tween-20 and 0.01% digitonin by pipetting up and down three times and incubated on ice for 3 min. After lysis, 1 ml of ATAC-seq RSB containing only 0.1% Tween-20 was added and the tubes were inverted to mix. Nuclei were then centrifuged for 5 min at max speed in a prechilled fixed-angle centrifuge. Supernatant was removed and nuclei were resuspended in 50 µl of transposition mix (25 µl 2X TD buffer, 2.5 µl transposase (100 nmol/L final), 16.5 µl PBS, 0.5 µl 1% digitonin, 0.5 µl 10% Tween20 and 5 µl water46) by pipetting up and down six times. Transposition reactions were incubated at 37°C for 30 min in a thermomixer with shaking at 1,000 r.p.m. Reactions were cleaned up with Qiagen MinElute columns. Libraries were amplified as described by Buenrostro et.al (3).

H3K27me3 ChIP-sequencing

Cells were washed with PBS and crosslinked with 1% paraformaldehyde (Thermo Fisher Scientific, 28906) for 10 min and quenched with 0.125 mol/L glycine for 5 min at room temperature. Crosslinked material was resuspended in 0.1% SDS (50 mmol/L Tris-HCl (pH 8), 10 mmol/L EDTA) and sonicated for 5 min with a Covaris E220 instrument (5% duty cycle, 140 Peak Incident Power, 200 cycles per burst, 1 ml AFA Fiber milliTUBEs). Soluble chromatin (5 µg) was immunoprecipitated with Protein A/G Dynabeads (Thermo Fisher Scientific, 10002D, 10004D) and 10 µg of H3K27me3 (Cell signaling, C36B11). ChIP-seq libraries were constructed using Accel-NGS 2S DNA library kit from Swift Biosciences. Fragments of the desired size were enriched using AMPure XP beads (Beckman Coulter). Libraries were sequenced on a NextSeq instrument (Illumina).

siRNA Experiments

Cells were transfected with 25 nmol/L siRNA pools (Dharmacon) using DharmaFECT 1 (Dharmacon). After 48 to 72 hours, cell viability was measured by CellTiter-Glo. Gene silencing efficiency was verified by immunoblotting and/or qPCR. The following SMARTpool ON-TARGET plus siRNA pools (Dharmacon) were used: Non-targeting siRNApool (D-001206-13-05), *EGFR* (L-003114-00-0005), *LIF* (L-011720-00-0005), *EZH2* (L-004218-00-0005).

In vitro drug screening studies

tSCLC, SCLC, LUAD cell lines were treated with 32 Drugs (Supplementary Table S7) either at 100 nmol/L or 1 μmol/L, and cell viability was measured after 120 h as described above. Drug response was assessed by calculating the ratio of cell viability of treated cells to vehicle treated cells.

1. Sholl LM, Do K, Shivdasani P, Cerami E, Dubuc AM, Kuo FC, et al. Institutional implementation of clinical tumor profiling on an unselected cancer population. *JCI Insight.* 2016;1(19):e87062.
2. Fisher S, Barry A, Abreu J, Minie B, Nolan J, Delorey TM, et al. A scalable, fully automated process for construction of sequence-ready human exome targeted capture libraries. *Genome Biol.* 2011;12(1):R1.
3. Buenrostro JD, Wu B, Chang HY, and Greenleaf WJ. ATAC-seq: A Method for Assaying Chromatin Accessibility Genome-Wide. *Curr Protoc Mol Biol.* 2015;109:21 9 1- 9 9.

Conflict of interest: PAJ has received consulting fees from AstraZeneca, Boehringer-Ingelheim, Pfizer, Roche/Genentech, Takeda Oncology, ACEA Biosciences, Eli Lilly and Company, Araxes Pharma, Ignyta, Mirati Therapeutics, Novartis, LOXO Oncology, Daiichi Sankyo, Sanofi Oncology, Voronoi, SFJ Pharmaceuticals, Biocartis, Novartis Oncology, Nuvalent, Eisai, Bayer, Transcenta, Silicon Therapeutics, Allorion Therapeutics, Accutar Biotech and Abbvie, Monte Rosa Therapeutics, Scorpion Therapeutics, Merus, Frontier Medicines, Hongyun Biotechnology, Duality Biologics, Blueprint Medicines, Dizal Pharma, GlaxoSmithKline, Tolremo, Myris Therapeutics, and Bristol Myers Squibb; receives post-marketing royalties from DFCI owned intellectual property on EGFR mutations licensed to Lab Corp; receives or has received sponsored research funding from AstraZeneca, Astellas, Daichi-Sankyo, PUMA, Boehringer Ingelheim, Eli Lilly and Company, Revolution Medicines, and Takeda; and has stock ownership in Gatekeeper Pharmaceuticals. DAB is a consultant for QIAGEN/N-of-One and Tango Therapeutics, has received research support from Bristol Myers Squibb, Novartis, Lilly, Gilead Sciences, and is a founder and shareholder in Xsphera Biosciences. MGO has received research support from Lilly Pharmaceuticals, Takeda Pharmaceuticals, Bristol Myers Squibb, and Novartis. CP has stock and other ownership interests in XSphera Biosciences, has received honoraria from Bio-Rad, is a consultant or in advisory role on DropWorks and XSphera Biosciences, has sponsored research agreements with Daiichi Sankyo, Bicycle Therapeutics, Transcenta, Bicara Therapeutics, AstraZeneca, Intellia Therapeutics, Janssen Pharmaceuticals, and Array Biopharma.
