# Supplementary material for: EGFR-mutant transformed small cell lung cancer harbors intratumoral heterogeneity targetable with MEK inhibitor combination therapy
Source: JCI Insight. 2026 Jan 23;11(2):e197008. doi: 10.1172/jci.insight.197008 (PMC12892894; doi:10.1172/jci.insight.197008)
Supplement: Unedited blot and gel images [file jciinsight-11-197008-s009.pdf]

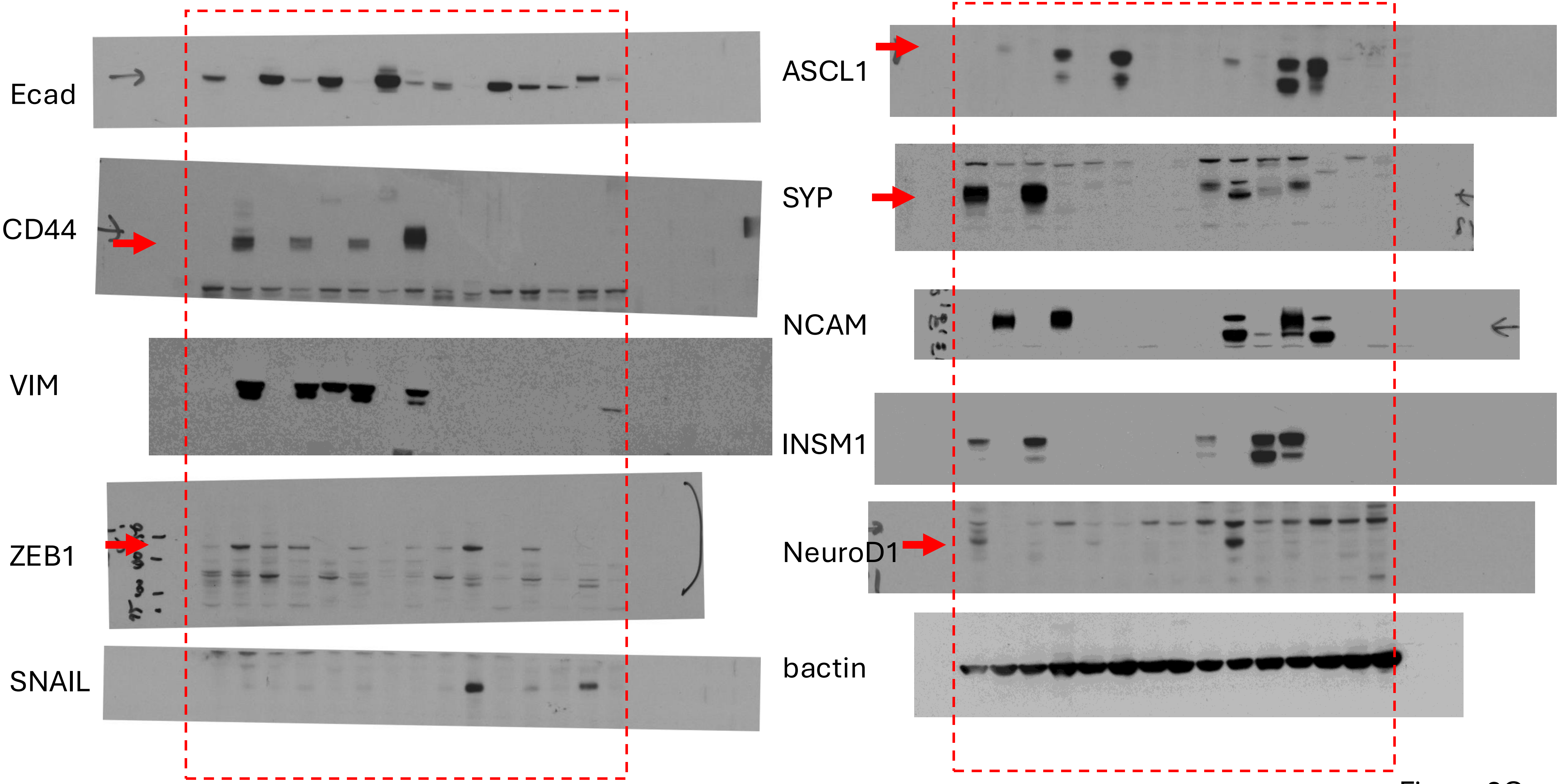
Ecad
CD44
VIM
ZEB1
SNAIL
ASCL1
SYP
NCAM
INSM1
NeuroD1
bactin

Figure 3C

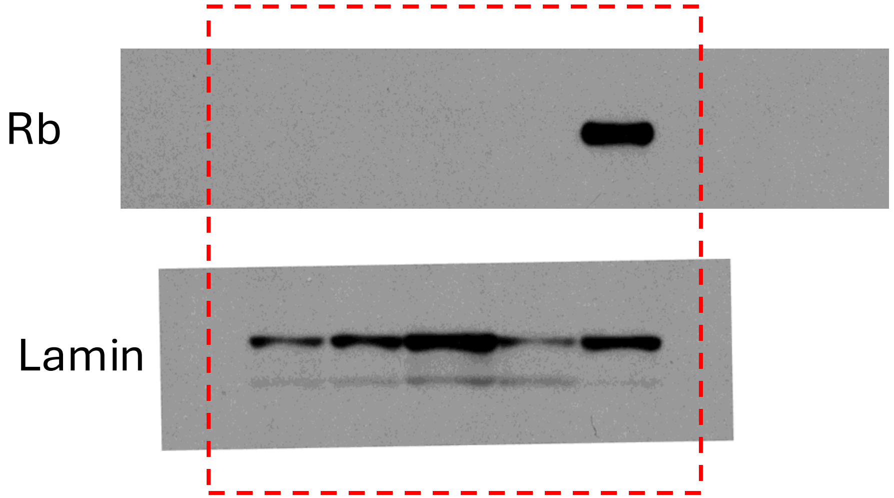

Figure 3D

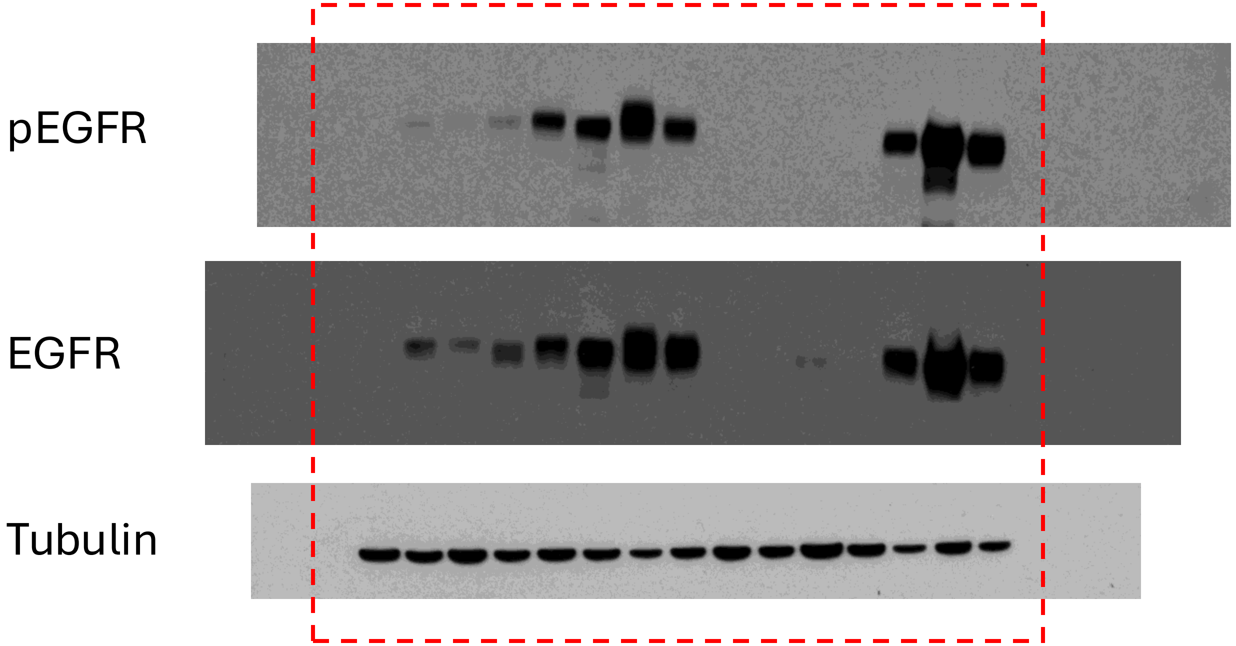

Figure 5A

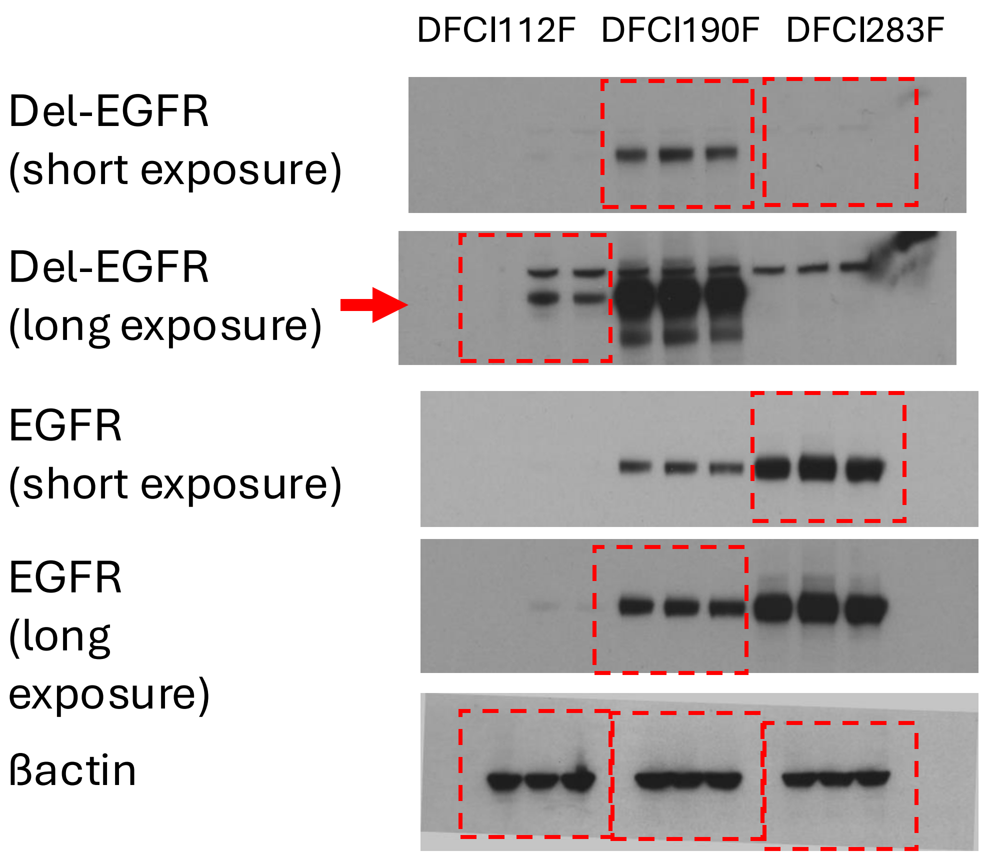

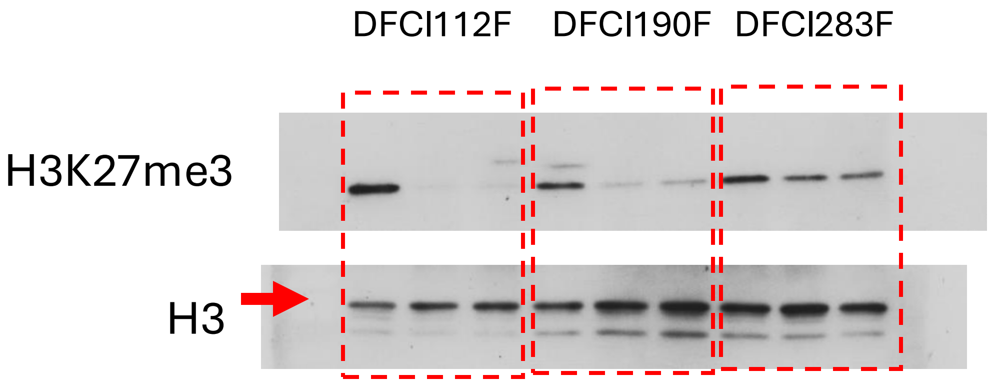

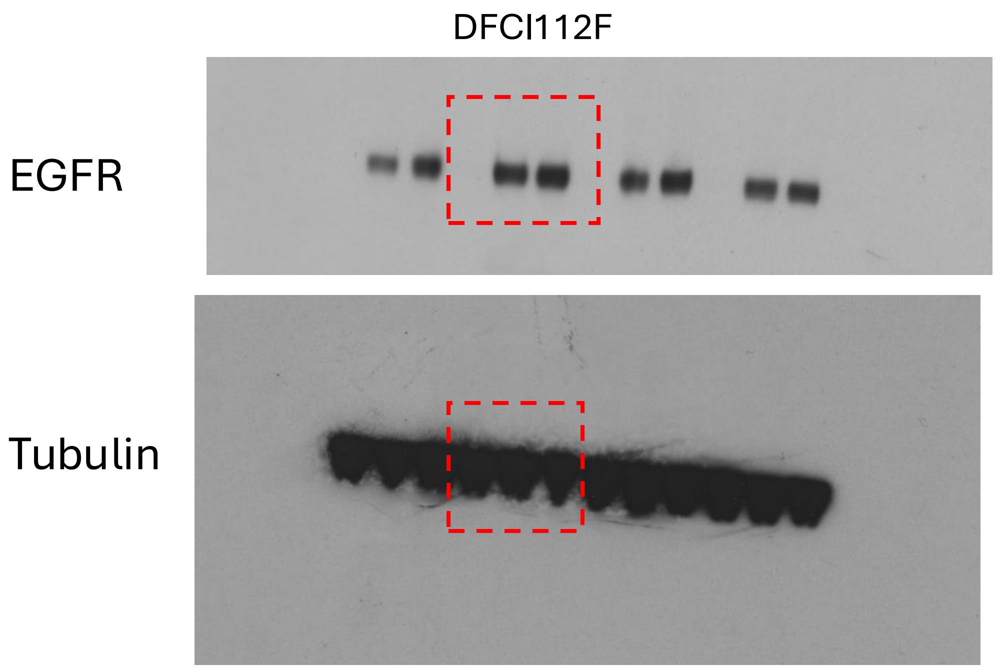

How the Experiment Was Performed: The western blots shown on the left were performed in parallel and run at the same time. For the EGFR blot of DFCI112, the signal was too weak using the standard protein loading amount (25 µg). Therefore, we repeated the experiment using the same cell line at a different passage number and increased the loading amount to 50 µg. This repeat experiment was performed at a different time and is shown on the right. Tubulin was used as the loading control for this experiment.

Figure 5B

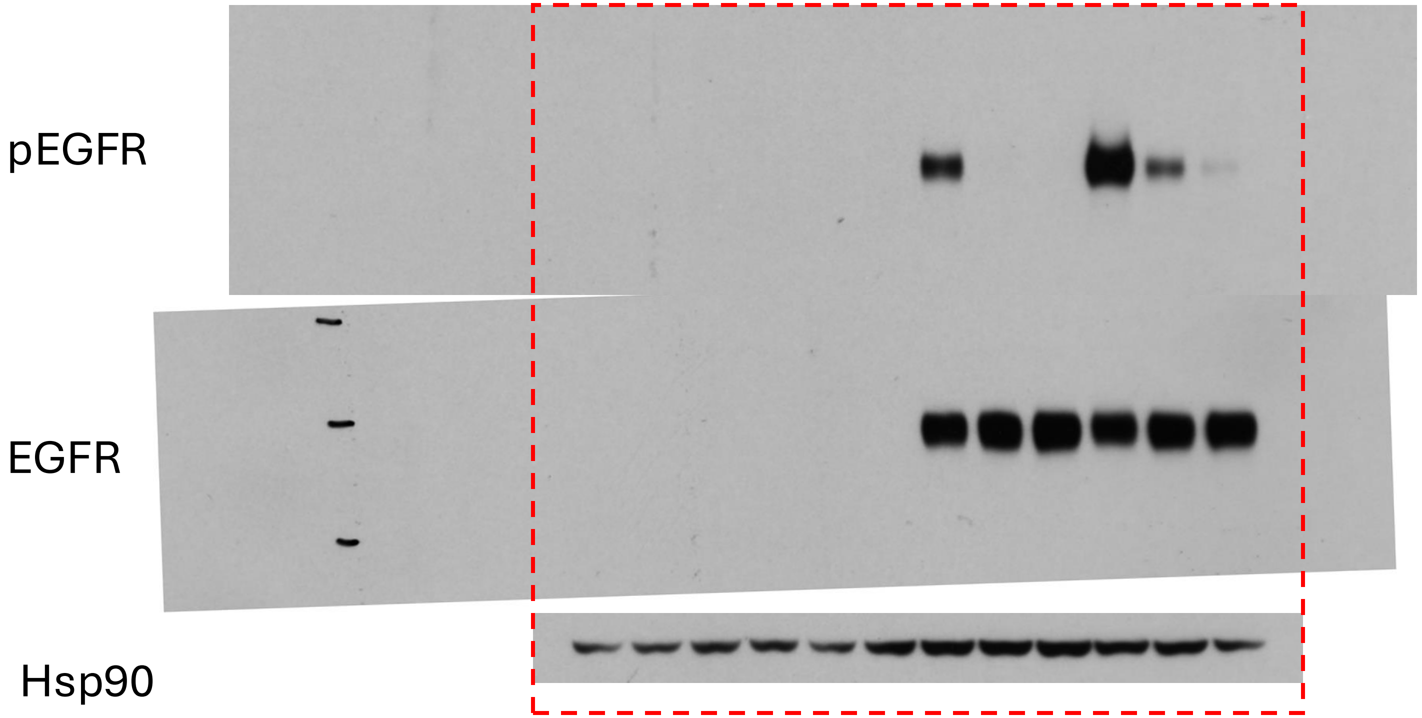

Figure 5E

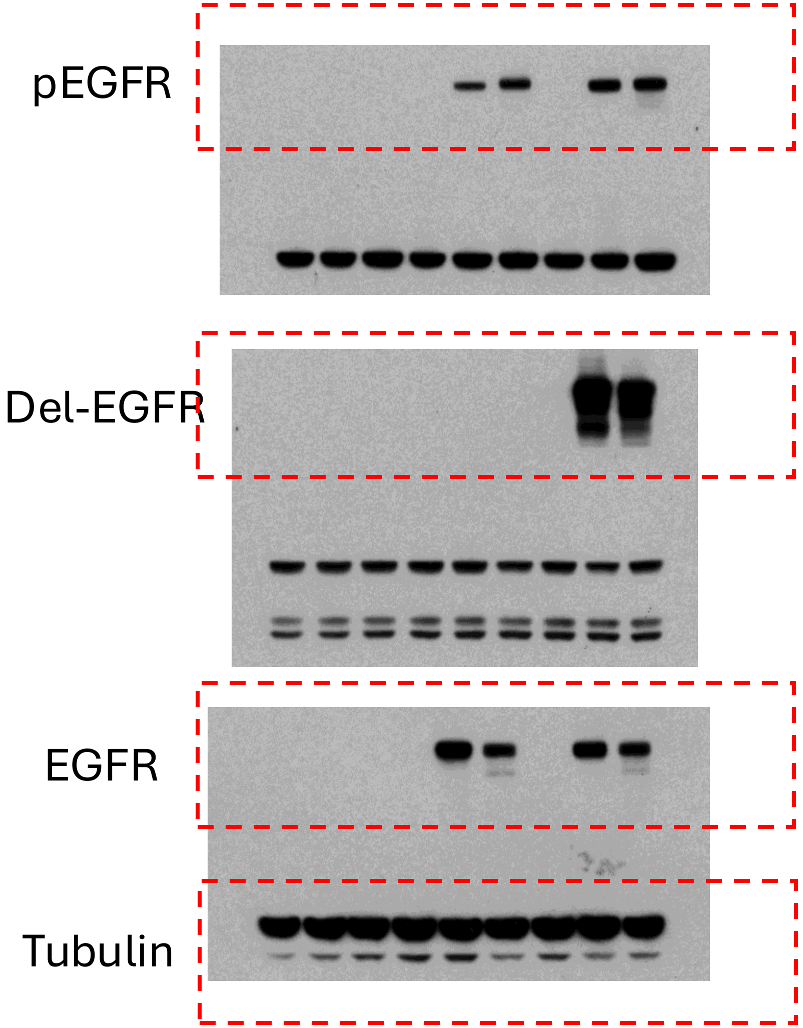

Figure 5G

DFCI112F

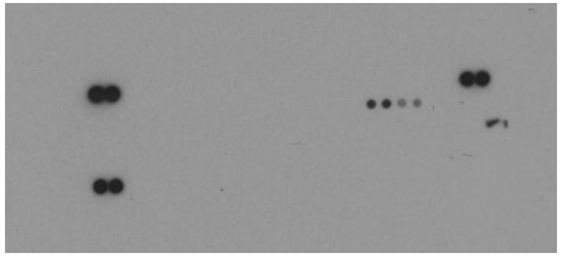

DFCI112Ad

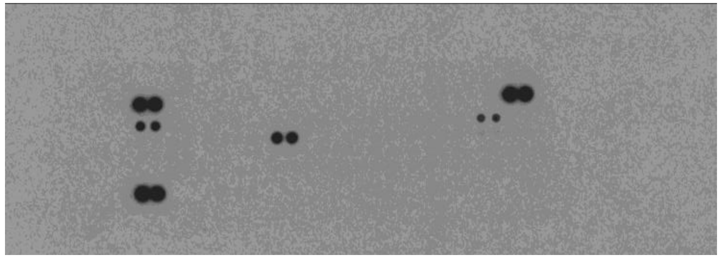

DFCI190F

DFCI190Ad

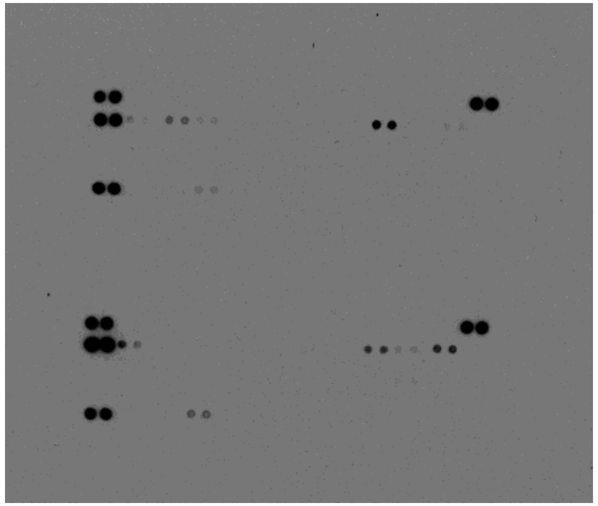

DFCI283F

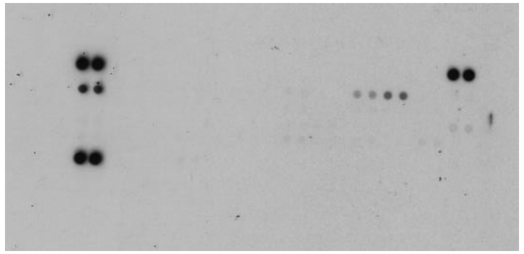

DFCI283Ad

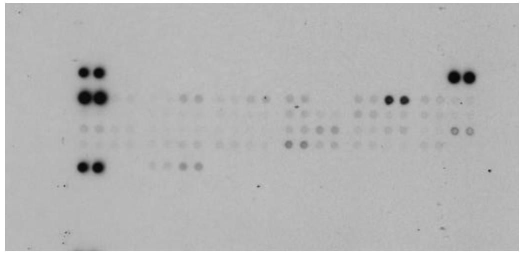

Supplementary Figure 8D

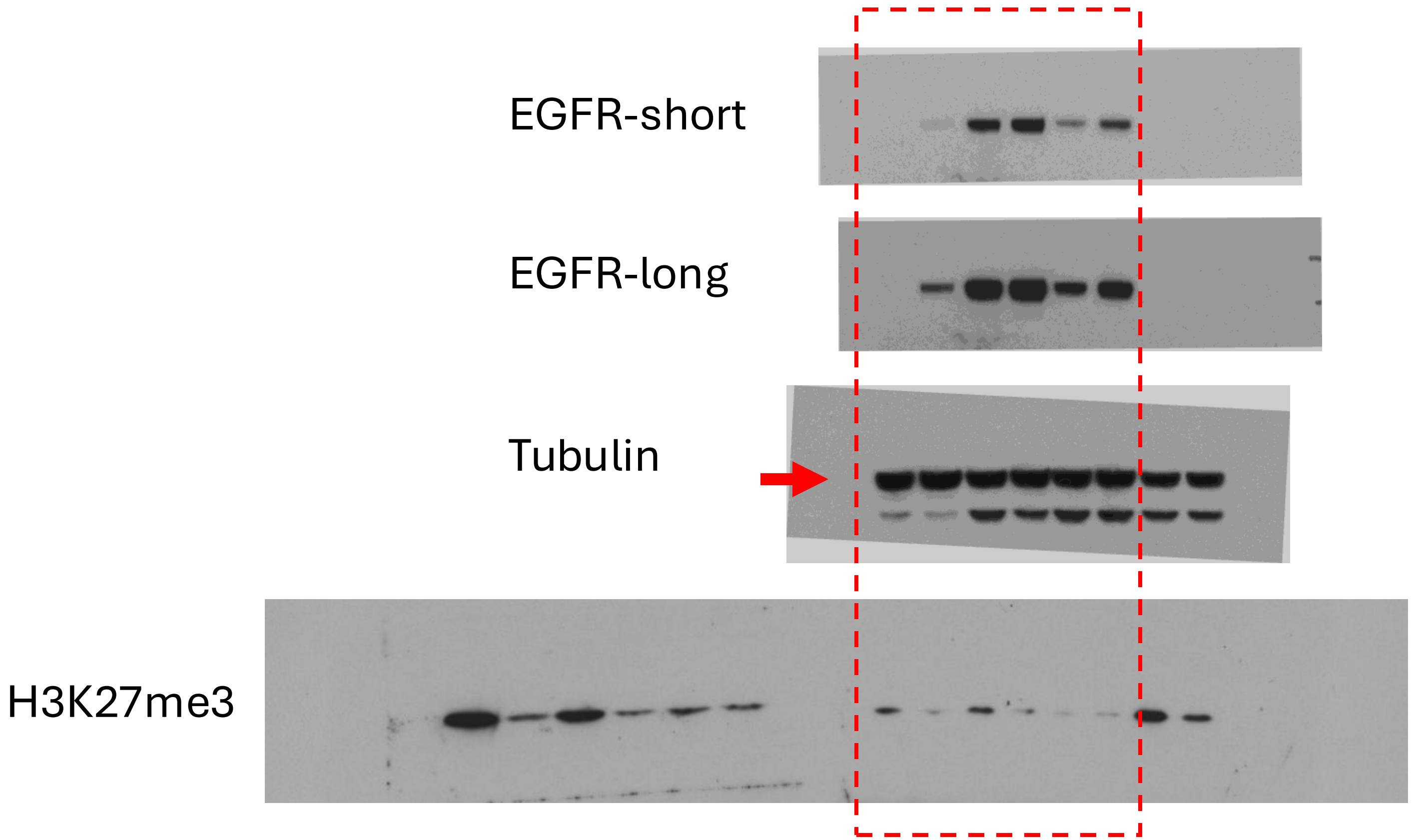

Supplementary Figure 8H

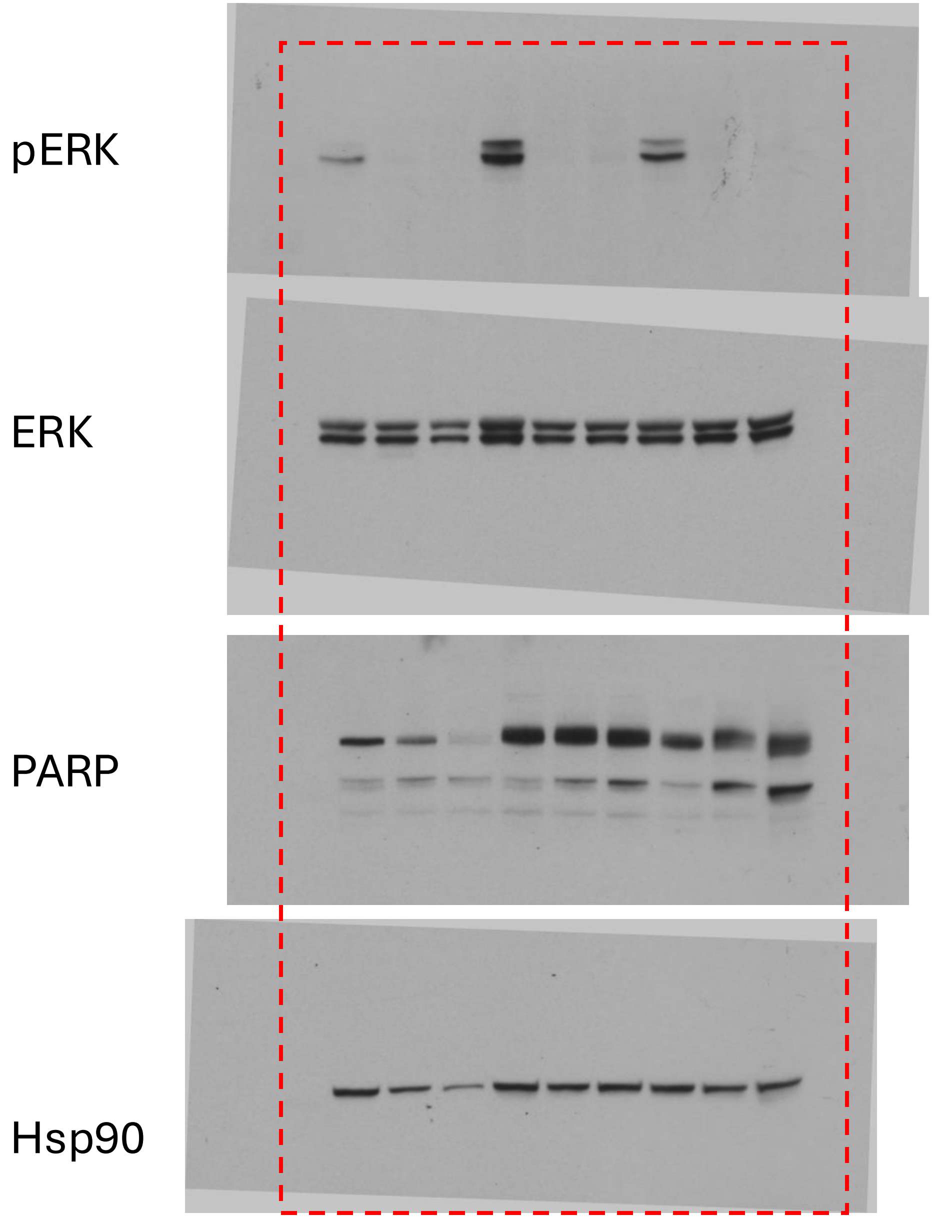

Supplementary Figure 9B
